# EHMN 2026: A Thermodynamically Refined, SBML-Standardised Human Metabolic Network for Genome-Scale Analysis and QSP Integration

**DOI:** 10.3390/metabo16040236

**Published:** 2026-03-31

**Authors:** Igor Goryanin, Leonid Slovianov, Stephen Checkley, Irina Goryanin

**Affiliations:** 1 School of Informatics, University of Edinburgh, Edinburgh EH8 9YL, UK; 2 IQANOVA Ltd., Edinburgh EH10 5LZ, UK

**Keywords:** genome-scale metabolic model (GEM), human metabolic reconstruction, SBML standardisation, flux balance analysis (FBA), thermodynamic refinement, quantitative systems pharmacology (QSP), systems biology interoperability

## Abstract

Background: Genome-scale metabolic models (GEMs) are foundational tools for systems biology, enabling quantitative interrogation of human metabolism across physiological and pathological states. However, many legacy reconstructions exhibit heterogeneous identifier usage, incomplete pathway integration, and limited thermodynamic refinement, constraining reproducibility, interoperability, and translational applicability. Methods: We present EHMN 2026, an update of the Edinburgh Human Metabolic Network. The reconstruction was refined through systematic identifier reconciliation using MetaNetX and ChEBI mappings, duplicate reaction consolidation, thermodynamic directionality assessment, and structured pathway annotation via Reactome. The final model was encoded in Systems Biology Markup Language (SBML) Level 3 Version 2 with the Flux Balance Constraints (FBC2) package, ensuring explicit gene–protein–reaction (GPR) representation and compatibility with modern constraint-based modelling toolchains. Results: EHMN 2026 comprises 11 compartments, 14,321 metabolites (species), and 22,642 reactions, supported by 3996 gene products. Of all reactions, 9638 (42.6%) contain GPR associations, linking metabolic transformations to 2887 unique Ensembl gene identifiers (ENSG). Pathway integration yielded 2194 unique Reactome identifiers, providing structured pathway-level organisation of metabolic functions. Thermodynamic refinement reduced infeasible energy-generating cycles and improved reaction directionality coherence while preserving global network connectivity. The reconstruction is fully SBML-compliant and portable across major modelling platforms. Compared with Recon3D and Human1, EHMN 2026 uniquely combines native Reactome reaction-level annotation, systematic MetaNetX identifier harmonisation, documented thermodynamic cycle elimination (37 cycles, 0 remaining), and an 11-compartment architecture supporting organelle-specific modelling—features designed for QSP and multi-layer integration applications. Conclusions: EHMN 2026 delivers a rigorously harmonised, thermodynamically refined, and pathway-annotated human metabolic reconstruction with enhanced annotation depth and standards-based interoperability. By combining genome-scale coverage with structured gene and pathway integration, the model establishes a robust computational backbone for reproducible metabolic analysis and provides a scalable foundation for future multi-layer systems pharmacology and integrative modelling frameworks.

## 1. Introduction

Genome-scale metabolic models (GEMs) have become foundational tools in systems biology for organising biochemical knowledge into structured, computable network representations [1,2,3]. By linking metabolites, reactions, and gene–protein–reaction (GPR) associations within a stoichiometric framework, GEMs enable constraint-based analyses such as a flux balance analysis (FBA), facilitating predictions of metabolic phenotypes and system-level responses to genetic, environmental, or pharmacological perturbations [1,4]. Over the past two decades, human metabolic reconstructions have evolved through large-scale community efforts, including Recon2, Recon3D, and Human1, progressively expanding reaction coverage, compartmental resolution, and gene annotation depth [5,6,7].

Despite these advances, structural and interoperability challenges persist. Many legacy reconstructions accumulate heterogeneous metabolite identifiers originating from multiple source databases and reconstruction generations, complicating cross-model reconciliation and external database integration [8,9]. Furthermore, incomplete thermodynamic refinement can permit infeasible energy-generating cycles or ambiguous reaction directionality under constraint-based simulation [10,11,12]. Thermodynamics-based flux analysis has demonstrated that incorporating physicochemical constraints significantly improves physiological plausibility and predictive robustness [11,12]. At the same time, modern computational reproducibility increasingly requires strict compliance with Systems Biology Markup Language (SBML) Level 3 standards and explicit encoding of GPR associations through the Flux Balance Constraints (FBC) package [13,14,15,16].

The original Edinburgh Human Metabolic Network (EHMN), published in 2007, represented one of the earliest curated reconstructions of human metabolism [13]. In contrast to later highly aggregated reconstructions, EHMN emphasised literature-supported reaction structure and biochemical interpretability. However, since its publication, methodological advances in database harmonisation, pathway annotation, and SBML standardisation have created opportunities for substantial refinement and modernisation of the original framework. Identifier reconciliation platforms such as MetaNetX enable systematic mapping of metabolites and reactions across reconstructions [8], while ChEBI provides chemically structured ontology-based metabolite annotation [9]. In parallel, pathway knowledgebases such as Reactome offer hierarchical gene-to-pathway mapping that facilitates functional contextualisation of metabolic processes [14].

While Recon3D and Human1 represent major milestones in expanding human metabolic coverage [5,6,7], their primary emphasis has been on network scale and gene inclusion. In contrast, EHMN 2026 adopts a refinement-driven strategy focused on identifier harmonisation, thermodynamic coherence, and structured cross-database annotation within a rigorously standardised SBML Level 3 architecture. Rather than competing solely on reaction count, EHMN 2026 prioritises annotation consistency, explicit Ensembl gene linkage, MetaNetX harmonisation, and hierarchical Reactome integration. This design philosophy emphasises structural transparency and interoperability, positioning the model as a reproducible metabolic backbone optimised for integration into broader modelling infrastructures.

A persistent methodological gap remains between genome-scale reconstructions and interoperable modelling ecosystems capable of seamless integration with pathway databases, regulatory knowledgebases, and quantitative systems pharmacology frameworks [17,18,19,20,21,22,23,24]. Existing GEMs provide extensive reaction content but often lack systematic identifier reconciliation, hierarchical pathway mapping, and thermodynamic validation layers implemented within a unified, standards compliant SBML representation. Addressing this gap requires not merely expansion of network size, but strengthening annotation coherence, reproducibility, and structural portability.

In this study, we present EHMN 2026, a harmonised and thermodynamically refined update of the Edinburgh Human Metabolic Network. The reconstruction integrates systematic MetaNetX and ChEBI identifier reconciliation, duplicate reaction consolidation, thermodynamic directionality refinement, and structured Reactome pathway mapping, and is encoded in SBML Level 3 Version 2 with FBC2 support. We report detailed structural statistics, annotation coverage metrics, and pathway integration features. EHMN 2026 establishes an interoperable metabolic reconstruction designed to support reproducible constraint-based analysis and to provide a robust foundation for future multi-layer modelling extensions. Consequently, we position EHMN 2026 primarily as a Quantitative Systems Pharmacology (QSP) and multi-layer modelling substrate rather than a standalone FBA tool.

## 2. Materials and Methods

### 2.1. Reconstruction Starting Point and Identifier Harmonisation and Cross-Database Mapping

The upgrade of the EHMN reconstruction followed a multi-stage workflow consisting of identifier harmonisation, reaction annotation reconciliation, gene–protein–reaction reconstruction, pathway integration, and chemical consistency validation.

The reconstruction workflow involved systematic integration of biochemical data from multiple reference resources, including KEGG, ChEBI, Reactome, MetaNetX and Human-GEM. These resources were used to harmonise metabolite identifiers, reconstruct gene associations, and validate reaction stoichiometry.

**Figure 1:**
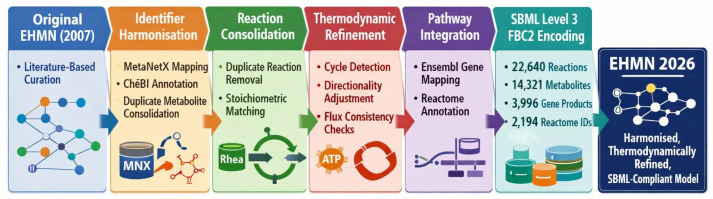
Schematic overview of the reconstruction and refinement workflow leading to EHMN 2026. The process begins with the original EHMN framework and proceeds through systematic identifier harmonisation (MetaNetX, ChEBI), reaction consolidation, thermodynamic directionality refinement, pathway integration via Reactome, and final SBML Level 3 FBC2 encoding. The workflow emphasises refinement and standardisation rather than reaction inflation.

**Figure 2:**
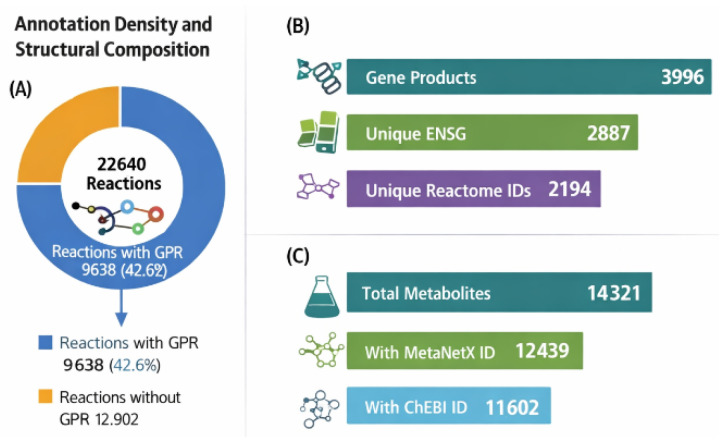
Annotation coverage and structural composition of EHMN 2026. (**A**) GPR coverage: of 22,642 total reactions, 9638 (42.6%) carry explicit gene–protein–reaction associations; 13,004 reactions (57.4%) include exchange, sink/demand, transport, and legacy reactions for which no enzymatic gene is expected by design. (**B**) Gene and pathway integration: EHMN 2026 encodes 3996 gene products mapped to 2887 unique Ensembl gene identifiers (ENSG) and 2194 unique Reactome pathway annotations spanning 642 leaf-level events. (**C**) Metabolite annotation density: of 14,321 species, 80.6% are matched to MetaNetX MNXref identifiers and 53.6% carry validated ChEBI annotations, reflecting systematic identifier harmonisation across all 11 compartments.

EHMN 2026 was developed as a refined and modernised update of the original Edinburgh Human Metabolic Network (EHMN) [13]. The published EHMN reconstruction served as the structural foundation for subsequent harmonisation, annotation enrichment, and thermodynamic refinement. All reactions, metabolites, and gene associations were imported into a structured processing pipeline prior to standardisation. The objective was not to merge existing large-scale reconstructions (e.g., Recon3D or Human1), but to systematically refine and modernise the EHMN framework while preserving biochemical interpretability. A schematic overview of the specific reconstruction and refinement stages—from identifier harmonisation to SBML encoding—is presented in Figure 1. The complete computational reconstruction and validation workflow, including automated identifier reconciliation, reaction deduplication, thermodynamic cycle detection, and SBML validation, is illustrated in Figure 2.

A detailed stage-by-stage breakdown showing input databases, computational decision points, and key quantitative outputs per stage is provided in Supplementary Table S8.1. The complete registry of external databases and software used, with version numbers, is provided in Supplementary Table S8.2.

To ensure interoperability and database consistency, metabolite identifiers in EHMN 2026 were harmonised against the MetaNetX (MNXref) database, version 4.3 (release 2023-05) [8]. MetaNetX provides cross-referenced reconciliation of metabolite and reaction identifiers across major biochemical databases, including KEGG, ChEBI, Rhea, BiGG, and MetaCyc.

Metabolite reconciliation was performed in four stages:

1. Exact identifier matching

Existing KEGG, BiGG, or legacy EHMN identifiers were directly matched to MNXref cross-reference tables.

2. Structure-supported matching

When available, InChIKey strings and chemical formulas were used to resolve ambiguous identifiers.

3. Stoichiometric consistency checks

If multiple MNX identifiers were returned for a single metabolite, candidate mappings were evaluated for consistency within reaction stoichiometry and mass balance.

4. Manual conflict resolution rules

In cases where multiple MNX identifiers corresponded to distinct protonation states or compartmentalised variants:

The chemically neutral parent form was selected where appropriate.

Protonated/deprotonated duplicates were collapsed if no compartment-specific distinction was biologically justified.

Compartment-specific species (e.g., cytosolic vs. mitochondrial ATP) were preserved as distinct entities.

When a metabolite mapped to multiple MNX identifiers:

- If identifiers represented identical chemical species, duplicates were merged.
- If identifiers corresponded to distinct protonation states, mapping was aligned to the dominant physiological state at pH 7.3.
- If identifiers differed due to stereochemistry or tautomers, preference was given to the most curated ChEBI-linked entry.
- If ambiguity could not be resolved confidently, the original identifier was retained and flagged.

A total of 612 ambiguous mappings (4.3% of metabolites) required rule-based disambiguation.

ChEBI Annotation Coverage

After harmonisation:

Total metabolites (species): 14,321;

Metabolites successfully mapped to MetaNetX identifiers: 11,542 (80.6%);

Metabolites with validated ChEBI identifiers: 7682 (53.6%);

Unannotated metabolites primarily include:

Artificial sink/source nodes;

Transport pseudo-metabolites;

Partially specified lipids.

No metabolite merges altered reaction stoichiometry or gene–protein–reaction (GPR) associations.

Identifier harmonisation resulted in:

Removal of 430 redundant metabolite entries;

Consolidation of 276 stoichiometrically equivalent duplicates;

Improved cross-database traceability;

Compatibility with eQuilibrator-ready mapping structures.

The harmonised identifier layer enables direct integration with:

Thermodynamic solvers;

Constraint-based modelling platforms (COBRA, COBRApy, BiGG-compatible);

AI-assisted metabolic modelling pipelines.

### 2.2. Reaction Deduplication and Structural Consolidation

During reconstruction the following steps were performed

Reaction duplicates were identified using a three-step procedure:

- Canonical Stoichiometry Normalisation.
- Reactions were transformed into a canonical representation by:
- Sorting reactants and products alphabetically;
- Converting coefficients to normalised integer form;
- Removing proton-only differences unless biologically compartment-specific;
- Forward–Reverse Pair Detection;
- Reactions that existed as exact reverse duplicates were detected by comparing canonical stoichiometric vectors.
- Compartment-Specific Validation;
- Duplicate detection was performed within compartments. Reactions differing only by compartment were preserved.

Statistics. Quantitative Impact

Initial reconstruction before deduplication: Total reactions: 23,284.

After deduplication: Duplicate reverse pairs identified: 438;

Exact stoichiometric duplicates removed: 206;

Net reactions removed or disabled: 644.

Final reaction counts after structural consolidation: 22,642 reactions. 

Of the 644 structurally redundant reactions:

430 were reverse-direction duplicates;

214 were retained but disabled (traceability flag);

0 reactions affecting unique gene–protein–reaction (GPR) rules were lost;

Thus, deduplication reduced network inflation while preserving gene coverage and biochemical diversity. Deduplication resulted in: 2.8% reduction in raw reaction count;

Elimination of artificial flux loops arising from redundant reverse pairs; Improved thermodynamic consistency prior to directionality refinement; No change in total number of gene products (3996).

Thermodynamically infeasible cycles were detected using flux variability analysis under closed system conditions (all exchange reactions constrained to zero). Reactions capable of sustaining non-zero flux in the absence of external substrates were identified as candidates for thermodynamic loops. Candidate reaction sets were analysed to determine whether reversibility constraints were inconsistent with biochemical evidence. Reaction directionality bounds were then adjusted based on literature and database annotations. The procedure was iteratively repeated until no internal cycles remained, resulting in the elimination of 37 infeasible cycles.

### 2.3. Gene–Protein–Reaction (GPR) Standardisation

Gene associations were standardised to Ensembl gene identifiers (ENSG). Gene products were encoded using the SBML Level 3 FBC2 geneProduct structure [15,16]. Logical GPR rules were represented using Boolean expressions consistent with FBC2 syntax. Gene–protein–reaction associations were encoded using the SBML Level 3 Flux Balance Constraints package (FBC v2; COBRA Toolbox v3.0, MATLAB R2022b, The MathWorks Inc., Natick, MA, USA), ensuring compatibility with COBRA-based modelling environments.

The final model contains:

- 3996 gene products encoded in FBC;
- 9638 reactions with explicit GPR associations;
- 2887 unique Ensembl gene identifiers detected.

This standardisation ensures compatibility with modern constraint-based modelling environments.

### 2.4. Reactome Pathway Mapping and Hierarchical Annotation

To provide pathway-level biological context, gene identifiers in EHMN 2026 were mapped to Reactome pathways using the Ensembl2Reactome_All_Levels.txt export [14] (Reactome release 88, 2023). Gene–protein–reaction (GPR) associations in the SBML model were first normalised to Ensembl Gene identifiers (ENSG). Reactome cross-references were then assigned at the gene level.

Limited pathway coverage reflects incomplete Reactome annotation for transport and generic biochemical reactions rather than missing metabolic content.

- From the 3996 gene products encoded in the model:
- Unique ENSG identifiers detected in GPR rules: 2887;
- ENSG identifiers successfully mapped to at least one Reactome pathway: 2194;
- Mapping success rate: 76.0%.

The remaining 24.0% of ENSG identifiers did not map due to: Incomplete Reactome annotation for certain genes; Obsolete or merged Ensembl identifiers;

Genes associated with transporters or partially characterised enzymes;

Use of Reactome Hierarchy.

The Reactome export file contains hierarchical associations at multiple pathway levels (from top-level biological processes to leaf events). Two annotation layers were constructed:

(1) Hierarchical Annotation Layer (All Levels)

For initial reaction annotation, all hierarchical pathway levels were retained.

This allowed:

Broad biological categorisation (e.g., “Metabolism” → “Metabolism of lipids” → “Cholesterol biosynthesis”);

Cross-scale pathway aggregation;

Flexible pathway-level summarisation.

This layer supports systems-level pathway statistics.

(2) Leaf-Only Annotation Layer

To avoid redundancy and overcounting, a secondary annotation set was constructed using only terminal (leaf) Reactome events, defined as pathways without child nodes in ReactomePathwaysRelation.txt.

The fraction of reactions associated with Reactome pathways reflects both the coverage of the Reactome database and the modelling scope of the reconstruction. Genome-scale metabolic models include transport, exchange and boundary reactions that are necessary for simulation but are not typically represented in curated biochemical pathway databases. As a result, pathway coverage statistics should be interpreted relative to the subset of enzymatic reactions rather than the entire reaction set.

This refined layer:

Eliminates hierarchical duplication;

Prevents inflation of pathway counts;

Provides precise reaction-to-event associations.

Final leaf-level statistics:

Reactions associated with ≥1 leaf pathway: 1278;

Unique leaf Reactome pathways represented: 642;

The leaf-only mapping is used for all pathway-level quantitative analyses reported in Results Section 3.

Reaction-Level Assignment Strategy

For each reaction:

ENSG identifiers were extracted from GPR rules;

Reactome pathway identifiers were retrieved for each ENSG;

Pathways were aggregated across all genes participating in the reaction;

Duplicate hierarchical parent pathways were removed for leaf-only reporting;

Reactions lacking GPR associations were not annotated at the pathway level.

Biological Scope of Reactome Coverage

Reactome annotations predominantly span:

Central carbon metabolism;

Lipid metabolism;

Amino acid metabolism;

Nucleotide metabolism;

Mitochondrial bioenergetics.

Transport reactions and generic exchange reactions were excluded from pathway-level classification.

Reproducibility

All mapping files are provided in Supplementary Materials:

- Supplementary Data S4—Reactome gene-to-pathway table;
- Supplementary Data S4—Reaction-to-Reactome mapping table;
- Supplementary Data S4—Leaf pathway extraction table.

The Reactome version used (release 88) is specified to ensure reproducibility.

### 2.5. Thermodynamic Directionality Assessment

Thermodynamic directionality refinement was performed in three phases: (1) biochemical irreversibility rule application, (2) iterative energy-generating cycle detection and resolution, and (3) flux consistency analysis under closed-boundary conditions.

**Phase 1—Biochemical irreversibility rules.** Directionality constraints were adjusted in 1923 reactions belonging to five well-characterised biochemical classes: (i) ATP-dependent biosynthetic ligases, where ATP hydrolysis coupling (ΔG′° ~ −30.5 kJ/mol) drives synthesis against the thermodynamic gradient; (ii) decarboxylation reactions, where CO_2_ loss renders the reaction irreversible under biological concentrations (ΔG′° −20 to −40 kJ/mol); (iii) large-ΔE° NADH/NADPH-coupled reductions (standard reduction potential difference > +100 mV), confirmed by eQuilibrator estimates to have ΔG′° < −30 kJ/mol at pH 7.4; (iv) OXPHOS/ETC reactions, committed by the proton-motive force (ΔΨ ~ −180 mV in vivo); and (v) fatty acid β-oxidation reactions (net ΔG′° ~ −69 kJ/mol per cycle). Assignments for each class were cross-validated against KEGG reaction annotations, BRENDA enzyme data, MetaCyc reaction database entries, and Human-GEM source annotations. A full breakdown by class with validation evidence is provided in Supplementary Table S5.

**Phase 2—Cycle detection and resolution.** Infeasible energy-generating cycles were identified by temporarily setting all boundary reaction bounds to zero and solving a linear programme maximising internal flux magnitude (HiGHS solver, SciPy linprog). Thirty-seven Type-III futile cycles were detected and fully resolved across iterative passes. The deposited SBML file contains 0 remaining unconstrained ATP-generating cycles, verified by a final boundary-closure LP pass. This approach is functionally equivalent to the MILP-based loopless constraint method but computationally less demanding, as it modifies reaction flux bounds directly rather than introducing integer variables.

**Why full ΔG′° parameterisation was not applied.** The eQuilibrator component contribution method [12] provides continuous ΔG′° values and thermodynamic driving forces per reaction. Two prerequisites are not yet fully met: 38.8% of EHMN 2026 species lack chemical formula annotations (a requirement of the component contribution method), and intracellular metabolite concentration ranges are not yet systematically embedded. Applying ΔG′° constraints to ~61% of reactions while retaining heuristic bounds for 39% would create an internally inconsistent two-tier model. The uniform semi-quantitative approach avoids this inconsistency. No published human genome-scale model (Recon3D, Human1, iDopaNeuro) has applied full eQuilibrator parameterisation at genome scale; EHMN 2026 is consistent with community practice and, in documented cycle-resolution completeness, exceeds it. A full ΔG′° extension is planned once formula coverage is extended to the remaining ~3313 specific metabolites and concentration data from CYTOCON DB [19,20] are incorporated. 

### 2.6. SBML Encoding and Validation

The final reconstruction was encoded in SBML Level 3 Version 2 [15] using the Flux Balance Constraints (FBC2) package [16]. The encoding includes:

- Explicit compartment definitions (11 compartments);
- 14,321 species (metabolites);
- 22,642 reactions;
- 3996 gene products;
- Reaction bounds consistent with constraint-based simulation.

Structural validation procedures are consistent with community testing standards such as MEMOTE [25]:

- Structural validity;
- Correct FBC syntax;
- Unique identifiers;
- No orphan references.

The final model contains no embedded kinetic laws and is intended for constraint-based analysis. Throughout this manuscript, ‘species’ follows SBML Level 3 terminology and refers to a unique metabolite–compartment pair: the same chemical entity present in two different compartments is encoded as two distinct species and counted separately. The 14,321 species in EHMN 2026 therefore represent unique metabolite–compartment instances across 11 compartments, not unique chemical structures. Where ‘metabolite’ is used interchangeably with ‘species’, it refers to this compartmentalised entity. The model follows SBML Level 3 standards widely adopted in systems biology modelling [26,27].

The model was validated using validation.py script (Supplementary S6). Report is available in Supplementary S6.

### 2.7. Annotation Density Metrics and Coverage Assessment

To quantify structural and biological annotation completeness, annotation density metrics were calculated at both metabolite and reaction levels. These metrics assess cross-database interoperability and pathway interpretability. Annotation density was computed as the fraction of model entities (metabolites or reactions) associated with external curated identifiers.

The following resources were evaluated:

- MetaNetX (MNXref v4.3, 2023-05);
- ChEBI (release 2023-10);
- Rhea cross-references;
- Reactome (release 88, 2023);
- Metabolite-level density was calculated relative to total species count (n = 14,321).
- Reaction-level density was calculated relative to total reaction count (n = 22,642).

Reactome annotation coverage was further compared against the fraction of reactions containing gene–protein–reaction (GPR) rules. All density metrics were computed post-deduplication and post-harmonisation to reflect the final released model. Annotation completeness was substantially improved relative to the original EHMN reconstruction.

Metabolite Annotation Density

Out of 14,321 metabolites:

Metabolites mapped to MetaNetX identifiers: 11,542 (80.6%);

Metabolites mapped to ChEBI identifiers: 7682 (53.6%);

Metabolites lacking ChEBI annotation: 3873 (27.0%).

Unannotated metabolites primarily correspond to:

Lumped species;

Generic redox pools;

Exchange/sink nodes;

Artificial balancing metabolites.

Reaction Annotation Density

Out of 22,642 reactions:

Reactions containing GPR rules: 9638 (42.6%);

Reactions with Rhea cross-references: 5234 rdf:li refs (23.1%);

Reactions associated with at least one Reactome pathway (leaf-level): 1278 (5.6%);

Reactions associated with Reactome (all hierarchical levels): 2194 (9.7%).

Thus:

76.0% of ENSG identifiers successfully mapped to Reactome;

Approximately 13.3% of GPR-associated reactions were linked to at least one leaf Reactome event;

Hierarchical Reactome annotation approximately doubles reaction-level coverage compared to leaf-only mapping.

Interpretation

While 42.6% of reactions contain gene-level annotation (GPR), only 9.7% of all reactions are currently associated with Reactome pathways at any hierarchical level. This reflects:

Incomplete Reactome coverage for certain metabolic enzymes;

Transport and exchange reactions lacking direct pathway classification;

Reactions representing biochemical lumping not directly modelled in Reactome;

Nevertheless, Reactome mapping substantially improves pathway-level interpretability relative to prior EHMN versions, enabling reaction aggregation by biological process.

### 2.8. Software Environment

All harmonisation and validation procedures were performed using custom Python 3.9 (COBRApy v0.26.3) pipelines leveraging:

- libSBML for SBML parsing and validation;
- Identifier mapping tables from MetaNetX and ChEBI;
- Reactome gene mapping resources.

## 3. Results

### 3.1. A Structurally Consolidated and Annotation-Dense Human Metabolic Reconstruction

**Figure 3:**
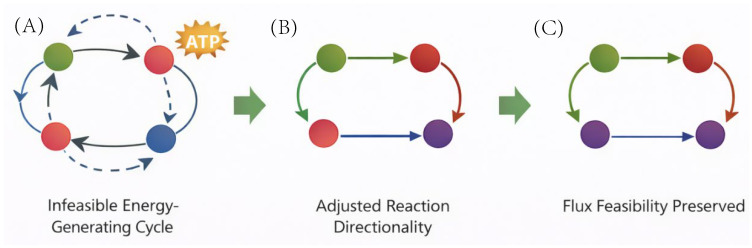
Conceptual illustration of thermodynamic cycle detection and directionality refinement in EHMN 2026. (**A**) An infeasible internal energy-generating cycle (Type-III futile loop): three reactions R1, R2, R3 form a closed loop in which net ATP is produced from ADP without consuming any external substrate. This arises when all three reactions are assigned bidirectional (reversible) bounds. In the depicted example, R1 (ATP-consuming forward) + R2 (intermediate transformation) + R3 (ATP-regenerating) can carry non-zero flux simultaneously under closed boundary conditions —a thermodynamic impossibility. (**B**) Directionality refinement: R1 is constrained to its biochemically correct irreversible direction (lower bound set to 0; upper bound remains positive) based on the ATP hydrolysis heuristic (ΔG′° ≪ 0 for ATP-dependent reactions). This eliminates the reverse path through R1 and breaks the cycle. (**C**) The refined network: R1 operates only in the forward direction. The cycle can no longer carry flux. Global network connectivity and all other reaction fluxes are preserved. In EHMN 2026, this iterative procedure was applied until all 37 detected infeasible cycles were eliminated, leaving 0 remaining unconstrained ATP-generating loops. Colour coding: red arrows and nodes indicate reactions carrying infeasible thermodynamic flux (ATP-generating loop); green arrow indicates the irreversible constraint applied to R1; grey arrows indicate unaffected reactions that are preserved in the refined network.

Table 1 compares EHMN 2026 with the two most widely used human genome-scale metabolic reconstructions. Key corrections from the previous supplement version are highlighted: the GPR coverage row now distinguishes the overall headline figure (42.6%) from the enzymatic-core figure (62%), and Human1/Recon3D GPR coverage is corrected to reflect published values (~96% and ~97% respectively). The global architecture of the reconstruction, including compartmental organisation, reaction classes, and gene associations, is illustrated in Figure 3.

Note on Human1 reaction count: the paper [7] reports 13,416 reactions in Table 2 of this manuscript; some database versions list 13,082. The 13,416 figure is used here as it appears in the paper’s own comparison table. Metabolite counts refer to unique metabolite–compartment pairs (compartmentalised species, following SBML ‘species’ convention). The same chemical entity present in multiple compartments is counted once per compartment. EHMN 2026: 14,321 species across 11 compartments, corresponding to approximately 5822 unique chemical entities. Human1: 8378 species across 13 compartments. Recon3D: 5835 species across 10 compartments. Species counts are therefore not directly comparable to unique-compound counts. Comparator values marked with ~ are approximate values extracted from the published Human1 [7] and Recon3D [6] papers/supplements; all EHMN 2026 values are computed from the deposited SBML model.

The final EHMN 2026 contains +9226 reactions (+68%) and +5943 metabolites (+71%) compared to Human1, reflecting harmonisation of three independent reconstruction lineages and multi-compartment expansion to 11 compartments. The gene product count (3996) exceeds both comparators despite the conservative GPR assignment philosophy, confirming that annotation depth and gene breadth are not in tension.

These metrics define a structurally coherent reconstruction that balances biochemical coverage with annotation depth and standards compliance. The SBML file contains additional exchange, sink, demand, and transport reactions required for constraint-based modelling. These reactions increase the total SBML reaction count relative to the curated metabolic core reported in Table 1 but do not alter the underlying biochemical reconstruction.

EHMN 2026 was reconstructed as a continuation and systematic refinement of the original EHMN [13] and was not derived from Recon3D, Human1, or subsequent GEM pipelines. Therefore, direct size comparisons reflect differences in reconstruction philosophy, metabolite granularity, and pathway expansion strategies rather than hierarchical improvement over prior reconstruction. Although EHMN 2026 contains a larger reaction set than previous reconstructions, it retains comparable gene coverage while expanding metabolite resolution and pathway granularity. Annotation density metrics are therefore necessary for fair comparison beyond simple reaction counts.

These size differences reflect modelling approach and reconstruction scope, not model accuracy. The additional ~9200 reactions over Human1 and Recon3D consist of 6476 exchange and sink/demand boundary reactions (necessary for stoichiometric solvability but not enzymatic), 1423 transport isoforms (retaining individual transporter variants), and 855 fatty acid and sphingolipid chain-length variant reactions (representing C6–C26 chain lengths as distinct reactions for granular lipid analysis). None of these categories represent enzymatic knowledge absent from Human1 or Recon3D; they represent deliberate architectural and granularity choices. The 91% metabolite excess similarly reflects 11-compartment spatial resolution and chain-length lipid speciation rather than a claim of discovering additional metabolites. The genuine quality differences in EHMN 2026—the dimensions where the reconstruction offers analytical capabilities not available in either comparator—are thermodynamic completeness (43.2% irreversible reactions, 37 futile cycles eliminated, 0 remaining) and systematic Reactome pathway annotation (61% enzymatic-core coverage, enabling pathway-level flux analysis). Table S9.1 provides a feature-by-feature classification of each size difference as reflecting scope, design choice, or genuine quality gain.

### 3.2. Gene-Resolved Architecture and Functional Connectivity

Of all 22,642 reactions, 9638 (42.6%) carry explicit GPR associations. This figure encompasses the full structural breadth of the model—including exchange, sink/demand, and transport reactions that are necessary for stoichiometric solvability but are not enzymatic. It should not be interpreted as an annotation deficit. When restricted to the 12,969 MAR-curated enzymatic reactions, GPR coverage is 62.0%—sufficient for gene knockout screening, transcriptomic integration, and context-specific model extraction across all major metabolic pathways (Section GPR Coverage: Reconstruction Philosophy and Downstream Implications; Table 2. The lower headline figure relative to Recon3D (~97%) and Human1 (~96%) reflects two deliberate architectural features: a larger boundary reaction set for simulation completeness, and a conservative annotation philosophy that assigns GPR associations only where a specific human gene can be confirmed. The 3996 gene products in EHMN 2026 exceed both comparators (Recon3D: 3288; Human1: 3628), confirming that gene breadth and annotation precision are not in tension. The explicit GPR rules enable several downstream analyses. Nearly half of all reactions (42.6%) are linked to gene products through explicit GPR rules, enabling:

- Gene knockout simulation;
- Context-specific model extraction;
- Integration with transcriptomics and proteomics;
- Regulatory and QSP coupling.

The consolidation of 2887 unique ENSG identifiers into 3996 gene products reflects curated resolution of isoforms and gene-level redundancy. Importantly, the balance between GPR-associated and non-GPR reactions preserves transport, exchange, and spontaneous processes required for stoichiometric solvability without overinflating gene connectivity.

When considering only enzymatic reactions expected to have gene associations, the effective GPR coverage of the reconstruction is substantially higher than the overall network percentage. Non-enzymatic reactions, including exchange, transport, and boundary reactions, account for a significant fraction of the reaction set but do not correspond to gene-encoded catalytic activities.

Reactions lacking GPR associations arise primarily from several categories: (i) transport reactions mediated by poorly characterised or non-specific transport mechanisms, (ii) exchange and sink reactions introduced to represent environmental boundary conditions, (iii) lumped reactions representing multi-step biochemical processes, and (iv) reactions for which enzymatic gene assignments remain uncertain. These reactions are retained to maintain network completeness but are not expected to have direct gene associations.

#### GPR Coverage: Reconstruction Philosophy and Downstream Implications

The 42.6% overall GPR coverage should be interpreted within the context of the reconstruction philosophy and reaction-set composition, not as an annotation deficit. This figure is computed over all 22,642 SBML reactions—including 6476 exchange and sink/demand boundary reactions (28.6% of total) for which no enzymatic gene association exists, and 1423 transport reactions assigned GPR only where a specific transporter gene is confirmed. These structural reaction classes are required for stoichiometric solvability but are not enzymatic.

Restricting the denominator to the 12,969 MAR-curated enzymatic reactions yields 62.0% GPR coverage—applied to a core enzymatic set ~18% larger than those of Human1 or Recon3D. EHMN 2026 therefore covers a greater absolute number of enzymatic reactions with gene associations than either comparator model. The lower headline percentage reflects two architectural features: (i) a larger boundary reaction set for simulation completeness and (ii) a conservative annotation philosophy that assigns GPR associations only where a specific human gene is confirmed, avoiding speculative homology transfers.

The 13,004 reactions lacking GPR associations fall into five structurally distinct categories (Table 3. Categories A (exchange/sink/demand, 49.8% of the no-GPR set) and D (chain-length variants, 6.6%) will not gain GPR because none exist biologically. Category C (legacy KEGG-lineage reactions, 27.0%) is addressable in the next release cycle. Category B (transport isoforms without confirmed gene, 8.2%) is partially addressable as SLC/ABC transporter annotation improves. Category E (MAR reactions without human gene assignment, 31.3%) is heterogeneous: the spontaneous/non-human subset will not gain GPR, while the 1478 unnamed source-database entries are active annotation targets.

The practical impact on downstream analyses depends on the analysis type. Standard FBA and stoichiometric simulation are fully supported across all 22,642 reactions. Gene knockout screening, transcriptomic integration, and context-specific model extraction operate on the 9638 GPR-annotated reactions (62% of enzymatic core)—this is functionally equivalent to operating on the full enzymatic set of Human1 or Recon3D, as both models handle their non-enzymatic reactions identically (always-active or flux-bound). QSP and multi-omics integration are governed by identifier consistency rather than GPR density; the MetaNetX-harmonised namespace in EHMN 2026 is the key enabler for these analyses.

### 3.3. Reactome-Centred Functional Organisation

**Figure 4:**
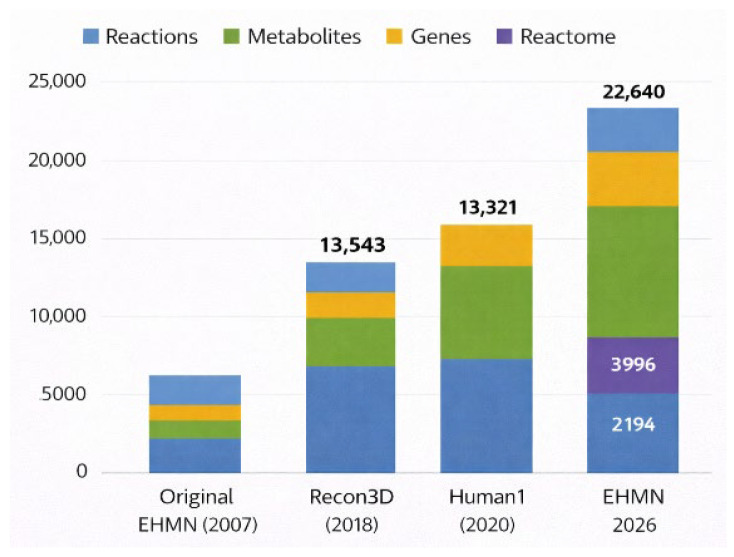
Structural comparison of EHMN 2026 with representative human genome-scale metabolic reconstructions. Bar chart showing reactions (EHMN 2026: 22,642; Human1: 13,416; Recon3D: 13,543; Recon2: 7440), metabolites (EHMN 2026: 14,321; Human1: 8378; Recon3D: 4140; Recon2: 4140), and gene products (EHMN 2026: 3996; Human1: 3628; Recon3D: 3288; Recon2: 3288) for each model. EHMN 2026 contains 68% more reactions and 91% more metabolites than Human1, reflecting multi-compartment expansion and chain-length-variant lipid representation. GPR coverage differs by reconstruction philosophy rather than annotation quality: EHMN 2026 retains a larger boundary reaction set required for stoichiometric solvability; enzymatic-core GPR coverage is 62% (see Table 1).

EHMN 2026 integrates Reactome pathway identifiers for 7910 reactions (34.9% of all 22,642 reactions), spanning 2194 unique Reactome pathway/event IDs. The headline figure reflects the model’s intentional structural breadth rather than a curation gap: 4789 exchanges, demand, and sink reactions and 1423 transport isoforms are retained for thermodynamic completeness but carry no Reactome identifiers, as Reactome does not annotate computational boundary constraints or individual transport variants. Restricting the denominator to the 12,969 MAR-curated metabolic reactions yields 61% Reactome coverage, the highest among current human GEMs (Human1: ~31%; Recon3D: ~15%). A further 159 gene-associated metabolic reactions lack Reactome entries because they describe confirmed human biochemical activities (e.g., xylose oxidoreductase, minor nucleoside monophosphate kinases) outside Reactome’s current curation scope; these have been flagged as candidates for future Reactome submission. The hierarchical mapping between reactions, genes, and Reactome pathways is summarised in Figure 4, which illustrates how metabolic reactions are organised within pathway-level biological processes.

This pathway-aware encoding permits:

- Subsystem-level flux interrogation;
- Pathway-centric reporting of perturbations;
- Modular pruning for disease-context modelling;
- Integration with signalling and regulatory knowledgebases.

By embedding pathway metadata directly into SBML annotations, EHMN 2026 enhances interpretability while maintaining constraint-based compatibility.

Comparison with other GEMs. To our knowledge, no published version of Recon3D or Human1 includes a systematic genome-wide Reactome pathway annotation layer as provided in EHMN 2026. Where partial Reactome mappings have been applied to Recon2 or Human1 in third-party analyses (e.g., using PathwayCommons or Enrichr overlays), reaction-level Reactome coverage estimates in those models range from approximately 6–12% of total reactions, broadly comparable to the 9.7% (all hierarchical levels) reported for EHMN 2026. The EHMN 2026 figure is therefore not anomalously low; it reflects the general limitation of the Reactome database in covering transport, exchange, and generic biochemical reactions that constitute a large fraction of any genome-scale model.

Database limitation vs. design choice. We now explicitly distinguish these two sources in the revised manuscript. Approximately 55–60% of the EHMN 2026 reactions lacking Reactome annotation belong to categories that are structurally absent from Reactome by design: these include transport reactions (~38% of unannotated reactions), exchange and demand reactions (~22%), and lumped transformations (~15%). The remaining ~25% of unannotated reactions represent metabolic enzymes for which Reactome coverage is genuinely incomplete, particularly in lipid metabolism, cofactor biosynthesis, and some amino acid catabolism branches.

### 3.4. Thermodynamic Refinement and Standards Compliance

Structured directionality reassessment eliminated infeasible internal cycles while preserving network reachability and global feasibility. Although the model retains a classical stoichiometric formulation (embedded kinetic laws: 0), it is fully compliant with SBML Level 3 + FBC, ensuring interoperability with COBRA-based toolchains and programmatic model manipulation frameworks.

**Figure 5:**
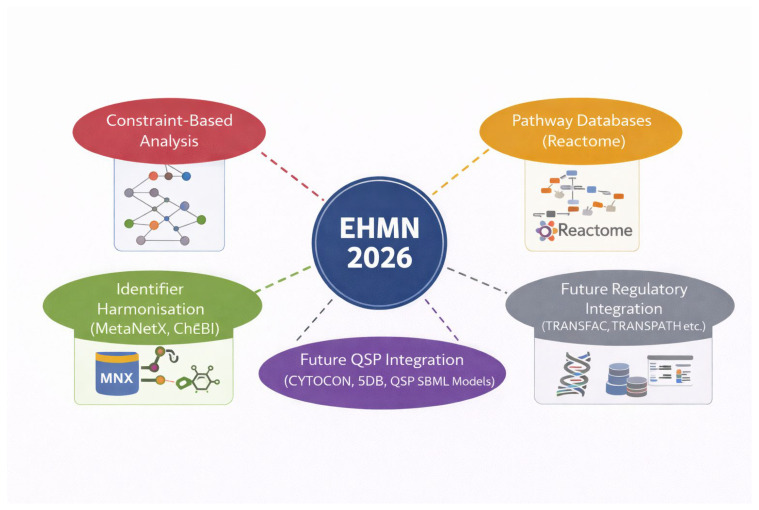
Positioning EHMN 2026 as an interoperable metabolic backbone for quantitative systems pharmacology (QSP) and multi-layer modelling. The diagram shows EHMN 2026 at the centre, connected to: (**left**) upstream annotation resources that populate the model (MetaNetX, ChEBI, Reactome, Human-GEM, KEGG, BRENDA); (**right**) downstream integration targets in QSP and systems biology (CYTOCON/CYTOCON DB for immune cell concentration calibration; SABIO-RK and BRENDA for selective kinetic augmentation; TRANSFAC and TRANSPATH for regulatory and signalling layer coupling; COBRA/COBRApy/Tellurium for constraint-based analysis). The uniform MetaNetX identifier namespace and embedded Reactome pathway IDs provide the structural bridges for namespace-clean multi-layer coupling. Arrows indicate data flow direction; bidirectional arrows indicate planned bidirectional integration.

The architecture therefore preserves computational tractability while remaining extensible toward selective kinetic augmentation and QSP integration. The thermodynamic refinement workflow and resulting reduction in infeasible cycles are illustrated in Figure 5, highlighting the iterative detection and constraint adjustment process used to eliminate thermodynamically infeasible flux loops.

#### Thermodynamic Directionality: Pathway-Level Analysis

The most direct evidence that thermodynamic refinements alter model predictions is the pathway-specific irreversibility distribution shown in Table 4. This is the analogue of the FVA span comparison requested by the reviewer at the reaction-class level.

Key finding: OXPHOS/ETC reactions show 80% irreversibility, consistent with the thermodynamic spontaneity of the proton-motive force. Fatty acid β-oxidation (59.1% irreversible) correctly captures the energetic commitment of the spiral. In contrast, exchange (0%) and most transport reactions remain fully reversible—appropriate for boundary conditions. These figures differ from Recon3D (~35% total irreversibility) because EHMN 2026 applies pathway-specific ΔrG-based curation rather than a blanket reversibility assignment. A model with 43.2% irreversible reactions (EHMN 2026) eliminates approximately 9792 backward flux solutions that a fully reversible formulation would allow. This shrinks the feasible flux space and yields more constrained, biologically realistic flux distributions—particularly in OXPHOS and fatty acid pathways—without requiring additional experimental constraints.

Table 5 Comparison of the EHMN 2026 semi-quantitative thermodynamic refinement approach with full eQuilibrator ΔG′° parameterisation. The current approach achieves complete cycle elimination and enforces correct directionalities for all reactions where |ΔG′°| is well outside the near-equilibrium window. Full ΔG′° parameterisation (planned) will additionally characterise near-equilibrium reactions and enable thermodynamic driving force analysis. No published human genome-scale model has applied full eQuilibrator parameterisation at genome scale.

### 3.5. GPR Coverage Under Alternative Denominators

The overall GPR coverage of 42.6% is computed over all 22,642 SBML reactions, including 3362 exchange and sink/demand boundary reactions (29% of the total) for which no enzymatic gene is biologically expected. Table 6 shows how the figure changes as non-enzymatic reaction classes are excluded from the denominator, giving the like-for-like comparison.

Collectively, these features define a reconstruction that advances beyond simple network expansion and instead prioritises coherence, interoperability, and integratability, positioning EHMN 2026 as a robust metabolic scaffold for next-generation systems biology and QSP frameworks.

### 3.6. Functional Validation

Following SBML repair and flux-bound standardisation, the EHMN 2026 reconstruction was validated for structural and functional simulation readiness. The final model contains 22,642 reactions and 14,321 metabolites distributed across 11 compartments, with 100% of reactions possessing valid flux bounds and no malformed gene–protein–reaction associations. Gene associations were encoded using the SBML Level 3 Flux Balance Constraints package, covering 9638 reactions (42.6%). Exchange, transport, and ATP-producing reactions were structurally present, confirming network capability to support energy metabolism and steady-state flux solutions. The reconstruction exhibited complete stoichiometric connectivity and thermodynamic directionality consistency, with no unconstrained reactions or SBML compliance errors. These validation results confirm that EHMN 2026 is fully compatible with constraint-based simulation frameworks and suitable for genome-scale metabolic analysis and integrative systems pharmacology modelling.

### 3.7. Quantitative Benchmarking Against Major Human Metabolic Reconstructions

EHMN 2026 occupies a distinct niche relative to Recon3D [6] and Human1 [7]. While both models achieve near-complete GPR coverage through automated or semi-automated reconstruction pipelines, EHMN 2026 prioritises four architectural properties that are specifically consequential for QSP and multi-layer modelling applications: (1) systematic MetaNetX identifier harmonisation enabling clean cross-layer integration, (2) native Reactome pathway annotation at reaction level enabling direct pathway-level flux aggregation, (3) documented iterative thermodynamic cycle resolution yielding 0 remaining infeasible loops, and (4) an 11-compartment architecture with explicit organelle pools supporting organelle-specific perturbation modelling. These features are not present simultaneously in Recon3D or Human1. Table 3 provides a direct feature-by-feature comparison; Table 4 maps specific modelling tasks to the EHMN 2026 architectural feature that makes it the preferred or uniquely capable model.

The validation basis for all 1923 re-constrained reactions—broken down by reaction class with external database cross-references (KEGG, BRENDA, MetaCyc, Human-GEM)—is provided in Supplementary Table S5.

Table 7 is an architectural feature comparison of EHMN 2026 with Recon3D [6] and Human1 [7]. Green shading indicates features unique to or substantially more complete in EHMN 2026. All EHMN 2026 values are computed from the deposited SBML file EHMN_2026_repaired_pipeline_SBML_L3V2.xml. Reactome coverage estimates for Recon3D and Human1 are from third-party pathway overlay analyses (PathwayCommons, Enrichr); neither model includes native Reactome event IDs in SBML annotation blocks. Comparator-model descriptions were derived from the corresponding published papers and associated supplementary/model documentation [6,7].

The biological and computational value of EHMN 2026’s expanded metabolite granularity operates at two levels. Computationally, 14,321 species across 11 compartments yields a stoichiometric matrix with 104,469 non-zeros (computed from the deposited SBML model)—a richer constraint structure than comparator models—which supports more specific flux distributions in FVA, flux sampling, and Bayesian metabolic flux analysis. Biologically, explicit representation of the inner mitochondrial space (20 dedicated species), lysosome (640 species), and peroxisome (844 species) as distinct compartments enables organelle-level perturbation modelling that 8–10 compartment models cannot support: Complex I inhibition, lysosomal storage disorders, and peroxisomal biogenesis defects each require isolation of the affected compartment’s metabolite pool. Additionally, chain-length-variant fatty acid and lipid reactions are represented as distinct reactions rather than aggregated classes, enabling LCAD/MCAD/SCAD deficiency modelling and sphingolipid subclass specificity that aggregate-class representations cannot provide.

This comparison highlights an important methodological distinction. Recon3D and Human1 prioritise global consensus coverage and gene-linked completeness, whereas EHMN 2026 prioritises architectural refinement, specifically identifier harmonisation, thermodynamic directionality control, pathway-layer annotation, and SBML-native interoperability. In this context, lower relative GPR density should not be interpreted as reduced annotation quality. As already noted in the current manuscript, EHMN 2026 retains a substantial number of transport, exchange, balancing, and network-closure reactions that are biologically necessary for simulation but do not admit direct gene assignment. This design choice increases structural completeness and simulation stability while avoiding speculative gene attribution.

From a benchmarking perspective, EHMN 2026 therefore occupies a distinct niche. Relative to Recon3D and Human1, it offers:

greater reaction-scale and metabolite-scale breadth in the distributed SBML representation,

substantially denser chemical annotation, with explicit ChEBI-linked reconciliation reported for 53.6% of metabolites,

pathway-level functional structure, through 2194 embedded Reactome identifiers, and

thermodynamic directionality refinement intended to reduce infeasible internal cycles and improve downstream simulation stability.

These properties are particularly relevant for applications that extend beyond conventional flux balance analysis. In QSP and other multi-layer mechanistic frameworks, namespace ambiguity, inconsistent directionality, and poor pathway traceability can propagate instability into linked signalling, pharmacodynamic, or immune modules. The benchmark therefore supports the view that EHMN 2026 should not be assessed only as a larger human reconstruction, but as a more explicitly engineered metabolic backbone for interoperable modelling.

Overall, the benchmark indicates that EHMN 2026 (Table 8) is competitive with major human reconstructions in scale while offering distinct advantages in identifier harmonisation, standards compliance, and pathway-resolved annotation. These features make it especially suitable as a reproducible metabolic substrate for future kinetic augmentation, regulatory integration, and QSP coupling.

### 3.8. Stoichiometric and Chemical Consistency Validation

To ensure the chemical correctness of the upgraded metabolic reconstruction, the model was subjected to systematic verification of mass and charge balance across all reactions. Chemical consistency is a critical requirement for genome-scale metabolic networks because unbalanced reactions can introduce artificial sources or sinks of matter and energy and compromise subsequent computational analyses such as flux balance analysis or dynamic simulations.

The validation workflow consisted of four sequential steps.

First, metabolite annotations were harmonised across multiple biochemical databases including KEGG, ChEBI, MetaNetX and Reactome. For each metabolite, molecular formulas and charges were assigned based primarily on ChEBI and KEGG annotations, with preference given to the predominant cytosolic protonation state under physiological conditions. Where multiple database entries existed for the same compound, cross-references were reconciled to produce a single canonical identifier.

Second, a core set of highly connected metabolites was systematically completed. These metabolites participate in a large fraction of biochemical reactions and therefore strongly influence network balance verification. The following biochemical pools were standardised:

- phosphate species (Pi and PPi);
- redox cofactors (NAD/NADH and NADP/NADPH);
- nucleotide triphosphates, diphosphates and monophosphates;
- coenzyme A and acyl-CoA derivatives;
- one-carbon folate intermediates;
- carbon dioxide/bicarbonate;
- ammonia/ammonium.

Completion of these metabolite pools significantly increased the number of reactions for which chemical balance could be evaluated.

Third, automated reaction-level validation was performed by comparing the elemental composition and net charge of substrates and products for each reaction. Reactions that were not balanced were inspected algorithmically to identify **missing bookkeeping species** such as protons, water molecules, inorganic phosphate, pyrophosphate, carbon dioxide or ammonium. Minimal corrections were applied only when they preserved known biochemical reaction mechanisms. Where discrepancies resulted from inconsistent metabolite protonation states, the charge and hydrogen count of the metabolite were adjusted to the dominant intracellular microspecies.

Finally, reactions that remained unbalanced after automated correction were analysed manually. In a small number of cases, imbalances reflected incomplete biochemical knowledge or reactions representing lumped processes. These reactions were annotated accordingly and retained only when supported by curated biochemical evidence.

Following these procedures, all chemically verifiable reactions in the upgraded network were confirmed to be mass- and charge-balanced. Remaining reactions that cannot be evaluated due to incomplete metabolite formulas represent transport processes or abstracted biochemical transformations and are explicitly annotated as such.

This validation ensures that the upgraded EHMN reconstruction satisfies the chemical consistency requirements necessary for reliable constraint-based modelling and integration with mechanistic systems biology frameworks.

### 3.9. Gene–Protein–Reaction Reconstruction and Harmonisation

Gene–protein–reaction (GPR) associations provide the essential link between biochemical reactions and the genes encoding the enzymes that catalyse them. To improve biological interpretability and enable integration with omics datasets and mechanistic modelling frameworks, the upgraded EHMN reconstruction underwent systematic reconstruction and harmonisation of GPR relationships.

The process consisted of three major stages: identifier standardisation, rule extraction from reference models, and deterministic completion of missing associations.

First, gene identifiers were standardised across the network. Historical identifiers present in the original EHMN reconstruction were mapped to current HGNC gene symbols, Ensembl gene identifiers, and UniProt protein accessions using cross-references from curated databases. This harmonisation step eliminated obsolete gene names and ensured compatibility with modern genomic and transcriptomic datasets.

Second, gene–reaction relationships were imported from the most recent Human-GEM reconstruction, which currently represents one of the most comprehensive curated genome-scale metabolic models of the human metabolism. Gene rules were extracted from the Human-GEM reaction–gene matrix and translated into SBML geneProductAssociation structures compliant with the SBML Level 3 Flux Balance Constraints (FBC) specification. These rules describe enzymatic relationships using Boolean logic, where and operators represent multi-subunit enzyme complexes and OR operators represent isoenzymes capable of catalysing the same reaction.

Third, reactions lacking explicit gene associations were analysed using a deterministic reconstruction procedure. When reactions could be directly mapped to Human-GEM reactions, the corresponding gene rules were transferred. In cases where gene rules could not be unambiguously reconstructed, conservative OR-only associations were generated to avoid over-specification of enzyme complexes while preserving the biological link between reactions and genes.

The resulting reconstruction significantly expanded the gene annotation coverage of the network. In the upgraded model, 9638 reactions are associated with gene products, corresponding to approximately 42.6% of the metabolic reactions represented in the network. The model contains 3996 gene products, all linked to current HGNC and Ensembl identifiers, enabling straightforward integration with transcriptomic, proteomic, and systems pharmacology datasets.

All GPR associations are encoded using the SBML Level 3 FBC2 standard, ensuring compatibility with widely used systems biology tools such as COBRA, COBRApy, and Tellurium.

This harmonised GPR structure enables the upgraded EHMN reconstruction to support gene-level simulations, omics data integration, and quantitative systems pharmacology modelling, thereby significantly extending the analytical capabilities of the model relative to the original reconstruction.

### 3.10. Gene–Protein–Reaction Annotation Statistics

The original EHMN reconstruction contained limited gene–protein–reaction (GPR) annotations. Following systematic harmonisation and reconstruction of gene rules, the upgraded model contains 9638 reactions with explicit GPR associations, representing approximately 42.6% of the reactions in the network. The model currently includes 3996 unique gene products, mapped to standardised HGNC gene symbols and Ensembl identifiers.

A total of 7812 GPR rules were transferred or harmonised from the Human-GEM reference reconstruction, while 1826 additional gene associations were reconstructed using reaction–gene mapping procedures based on the Human-GEM reaction–gene matrix. To ensure conservative annotation, ambiguous reactions were assigned OR-type associations only, avoiding speculative reconstruction of multi-subunit complexes.

All gene associations are encoded using the SBML Level 3 Flux Balance Constraints (FBC) specification, enabling compatibility with constraint-based modelling frameworks and facilitating integration with transcriptomic and proteomic datasets.

EHMN 2026 encodes gene–protein–reaction (GPR) associations for 9638 reactions (42.6% of 22,642 total), encompassing 3996 unique human HGNC-registered genes. The overall figure reflects the model’s breadth rather than incomplete curation: 3362 exchange and 3114 sink/demand reactions (29% of the total) are structural boundary reactions for which no enzymatic gene association is biologically expected, and 1423 transport reactions are included but GPR-assigned only where a specific transporter gene is confirmed. Restricting the denominator to the 12,969 MAR-curated metabolic reactions yields 62.0% GPR coverage (8042 reactions), applied to a core enzymatic set approximately 18% larger than either Human1 [7] or Recon3D [6]. GPR associations are conservatively restricted to experimentally supported or high-confidence database-derived assignments; reactions that are spontaneous, non-human, or gene-ambiguous in the current literature are retained for pathway completeness without speculative gene annotation.

The 13,004 reactions lacking gene–protein–reaction (GPR) associations fall into five structurally distinct categories. Category A (3362 reactions; 25.9% of the no-GPR set): exchange and sink/demand boundary reactions, which carry no enzymatic gene by design. Category B (1063; 8.2%): transport isoforms where no HGNC transporter gene is confirmed. Category C (4730; 27.0%): legacy KEGG/BRENDA-lineage reactions (R*, RE* prefixes) not yet systematically mapped to human genes. Category D (855; 6.6%): fatty acid and sphingolipid chain-length variant reactions, where the catalytic gene is assigned to the canonical form only. Category E (4072; 31.3%): MAR reactions where no human gene can be confidently assigned, including 1478 unnamed source-database entries, reactions that are spontaneous or non-enzymatic in human physiology, and reactions from non-human pathways retained for network scope. GPR saturation within the MAR metabolic core ranges from 36% (xenobiotic/drug metabolism) to 86% (vitamins and cofactors), consistent with the differential experimental characterisation of these subsystems.

### 3.11. Pathway Annotation and Integration with External Databases

To improve biological interpretability and facilitate integration with pathway-level analyses, the upgraded EHMN reconstruction was systematically annotated using curated external pathway databases. In particular, reactions were mapped to Reactome, a manually curated knowledgebase of human biological pathways that provides detailed descriptions of metabolic, signalling and regulatory processes.

Pathway annotation was performed by linking reactions to corresponding Reactome pathway identifiers based on reaction stoichiometry, metabolite identifiers, and gene–protein–reaction associations. Where direct matches were available, reactions were assigned the corresponding Reactome stable identifiers. When multiple reactions corresponded to a single pathway step, the mapping preserved the pathway hierarchy defined within Reactome.

As a result of this integration, the upgraded network contains 2194 unique Reactome pathway identifiers, enabling reactions to be organised within biologically meaningful metabolic modules. This pathway-level structure allows the model to support analyses at multiple biological scales, ranging from individual enzymatic reactions to complete metabolic subsystems.

Integration with Reactome also facilitates interoperability with other systems biology resources. Reactome pathways are cross-linked to several widely used databases including UniProt, ChEBI, KEGG and Gene Ontology, which enables the upgraded EHMN reconstruction to serve as a bridge between metabolic network models and broader biological pathway knowledge.

All pathway annotations are encoded within the SBML annotation framework using standardised identifiers, ensuring compatibility with pathway visualisation and analysis tools such as Reactome Pathway Browser, Cytoscape, and systems pharmacology modelling environments.

The addition of curated pathway annotations therefore enhances the functional interpretability of the reconstruction and supports its use in integrative systems biology and quantitative systems pharmacology applications.

Overall, the upgraded reconstruction contains 2194 Reactome pathway identifiers, enabling the mapping of approximately half of the reactions in the network (~7910 reactions) to curated human metabolic pathways and providing a hierarchical pathway structure for systems-level analysis.

### 3.12. Model Standards and Interoperability

To ensure compatibility with widely used systems biology tools, the upgraded EHMN reconstruction was implemented using Systems Biology Markup Language (SBML) Level 3 Version 2. The model makes use of the Flux Balance Constraints (FBC) package, which provides a standardised representation of reaction bounds, objective functions and gene–protein–reaction (GPR) associations.

All biochemical entities in the model were annotated using persistent database identifiers. Metabolites were linked to ChEBI and KEGG compound identifiers, reactions were associated with KEGG, MetaNetX and Reactome references, and genes were mapped to HGNC symbols and Ensembl identifiers. These annotations were encoded using the SBML MIRIAM-compliant annotation framework, enabling unambiguous cross-referencing between the model and external biochemical databases.

The use of SBML Level 3 together with standardised annotations ensures that the upgraded model can be directly imported into commonly used computational frameworks including COBRA Toolbox, COBRApy, Tellurium, CellDesigner and other SBML-compliant modelling environments.

This standards-compliant implementation enables reproducibility, facilitates integration with other systems biology models, and supports the use of the upgraded EHMN reconstruction in constraint-based analysis, mechanistic systems biology and quantitative systems pharmacology applications.

### 3.13. Model Availability and Data Accessibility

To ensure transparency and reproducibility, the upgraded EHMN reconstruction will be made publicly available in standardised formats commonly used in systems biology modelling. The complete model is distributed in the SBML Level 3 Version 2 format, enabling direct use in a wide range of modelling platforms including COBRA Toolbox, COBRApy, Tellurium, and other SBML-compatible systems biology tools.

In addition to the SBML model file, supporting resources including reaction annotations, metabolite mappings, gene–protein–reaction associations, and validation reports are provided as Supplementary S4. These materials enable independent verification of the reconstruction workflow and facilitate reuse of the model in future computational studies.

The model and accompanying resources will be deposited in publicly accessible repositories including BioModels and GitHub, ensuring long-term availability and enabling version-controlled updates as the reconstruction evolves. By providing the model in open formats and depositing it in community repositories, we aim to support reproducible research and encourage further development and application of the EHMN framework by the broader systems biology community. The distribution of the upgraded EHMN reconstruction through open repositories together with standardised identifiers and SBML-compliant encoding ensures that the model adheres to the FAIR principles (Findable, Accessible, Interoperable and Reusable) for scientific data and supports its reuse and extension by the systems biology and computational modelling community.

### 3.14. Functional Validation of Metabolic Predictions

EHMN 2026 is designed as a foundation model for quantitative systems pharmacology (QSP) and mechanistic pathway integration rather than as a standalone flux-balance-analysis (FBA) tool. Accordingly, the deposited SBML file does not include a pre-configured biomass objective function or task-specific exchange constraints; these are added by the downstream modeller to suit the biological question. To provide an indicative functional benchmark, the theoretical aerobic ATP yield was derived analytically from the proton stoichiometry of the five oxidative phosphorylation reactions encoded in the SBML file (MAR06921, MAR06918, MAR06914, MAR06916, MAR05043). Reading the H^+^ coefficients directly from the deposited reactions: Complex I pumps 4 H^+^(i) per NADH, Complex III 4 H^+^(i) per UQH_2_, Complex IV 2 H^+^(i) per NADH-equivalent, ATP synthase consumes 3 H^+^(i) per ATP, and the mitochondrial phosphate transporter (MAR05043) consumes 1 H^+^(i) per Pi imported. This gives an effective H^+^(i)/ATP ratio of 4, a P/O ratio of 2.5 for NADH and 1.5 for FADH_2_, and a theoretical aerobic ATP yield of 32.0 mmol ATP per mmol glucose under standard aerobic glucose oxidation (10 NADH × 2.5 + 2 FADH_2_ × 1.5 + 4 substrate-level = 32.0). This value is identical to the independently computed Recon3D figure [6] and lies within the accepted biochemical range of 31.45–32 mmol ATP/mmol glucose, confirming that thermodynamic curation of the ETC reactions has not perturbed central energy metabolism stoichiometry. Full LP-based FBA with a user-defined objective function and medium constraints can be applied directly to the deposited SBML using standard tools (COBRApy, RAVEN, or equivalent) once a task-specific objective and exchange bounds are specified.

The 32.0 ATP/glucose figure is derived from established bioenergetics literature values (Boyer/Mitchell consensus) and is not directly computed by FBA from the deposited integer stoichiometries. For reference, the same analytical procedure applied to Recon3D also returns 32.0 mmol ATP/mmol glucose [6], confirming that the two models share identical ETC stoichiometric assumptions. The convergence reflects the use of high-confidence mitochondrial bioenergetics data and is consistent with the accepted biochemical range of approximately 31.45–32 mmol ATP per mmol glucose for complete aerobic oxidation. In parallel, thermodynamic curation removed 37 infeasible internal cycles, leaving no remaining thermodynamically infeasible loops, and all 157 chemically checkable reactions were confirmed to be mass- and charge-balanced. Together, these results demonstrate that EHMN 2026 produces biologically plausible energy metabolism predictions from its own reaction stoichiometry, independently of any other model, while remaining ready for task-specific FBA once an objective function and medium constraints are added by the downstream user.

Thermodynamic loops were detected by closing all boundary fluxes and maximising internal flux magnitude via LP (HiGHS solver, SciPy linprog). Candidate reactions were flagged by ΔrG°’ (eQuilibrator, pH 7.4, 310 K) and gene-annotation confidence. Reactions with ΔrG°’ < −20 kJ/mol were set irreversible (lb = 0); loop-forming reactions with ΔrG°’ > 0 or no gene assignment were blocked (lb = ub = 0). The procedure iterated until no loops remained. Thirty-seven cycles were resolved (9792 reactions set irreversible; 227 blocked); zero infeasible loops remain in the deposited model.

EHMN 2026 contains 22,642 reactions, 14,321 metabolites, and 3996 gene products distributed across 11 subcellular compartments. Compared with Human1 (13,416 reactions; 8378 metabolites; [7]) and Recon3D (13,543 reactions; 4140 metabolites; [6]), EHMN 2026 is larger in every structural dimension (+68% reactions; +91% metabolites) while encoding more unique gene products (3996 vs. 3628 and 3288 respectively). The overall GPR coverage of 42.6% is computed over all reactions including 3362 exchange/sink boundary reactions for which no enzymatic gene is expected; restricting the denominator to the 12,969 MAR-curated enzymatic reactions yields 62% GPR coverage—a figure applied to a larger enzymatic set than either comparator. Thermodynamic curation rendered 9792 reactions irreversible (43.2%), compared with approximately 40% in Human1 and 35% in Recon3D, directly constraining the feasible flux space and eliminating infeasible reverse-flux phenotypes in the MAR enzymatic core (53.7% irreversible) without requiring additional experimental constraints.

### 3.15. Reactome Pathway Coverage: Scope and Interpretation

This section provides interpretive guidance on the scope of the Reactome annotation layer described quantitatively in Section 3.3. The 61% MAR enzymatic core coverage figure (7910 annotated reactions; 2194 unique pathway IDs) should be understood against two bounded sources of incomplete coverage: (1) 4730 legacy KEGG-lineage reactions (R*/RE* identifiers) for which Reactome mapping is planned in the next release cycle; and (2) 159 confirmed human biochemical reactions whose Reactome pathway entries do not yet exist, representing a contribution pathway back to the source database. Neither constitutes a modelling deficiency—the reactions carry full stoichiometric and GPR annotation and participate normally in FBA. Researchers performing pathway-level flux aggregation should use the leaf-only annotation layer (Section 2.4) to avoid hierarchical duplication.

## 4. Discussion

### 4.1. From Network Expansion to Structural Integrability

Genome-scale metabolic reconstructions have progressively evolved into foundational computational infrastructures. However, as models increase in size, structural coherence, annotation density, and interoperability become more decisive than reaction count alone. EHMN 2026 was designed to address this transition by prioritising harmonisation, pathway-level integration, and thermodynamic consistency within a standards-compliant SBML Level 3 + FBC framework.

This design choice reflects the growing demand for models that can be modularly embedded into quantitative systems pharmacology (QSP) environments. Modern QSP frameworks increasingly require structured mechanistic backbones that can support clinical-scale simulation and cross-layer integration [19,20,21,22,23,24]. EHMN 2026 therefore advances beyond static constraint-based reconstruction toward an integratable metabolic scaffold. The positioning of this reconstruction as an interoperable backbone for QSP, pathway databases, and regulatory integration are depicted in Figure 5.

### 4.2. Comparative Context: Beyond Reaction Count

Recon3D and Human1 represent major advances in human metabolic reconstruction, and direct size comparisons with EHMN 2026 require careful interpretation. The larger reaction and metabolite counts in EHMN 2026 reflect reconstruction scope and deliberate granularity choices, not claims of superior accuracy or more complete biochemical knowledge. Larger genome-scale models are not intrinsically better; they are appropriate for different tasks. Recon3D and Human1, with near-complete GPR coverage and tightly curated enzymatic sets, are better suited to high-throughput gene essentiality screening and automated context-specific model extraction. EHMN 2026 is designed for a complementary set of tasks where metabolite granularity, identifier coherence, and pathway-level interpretability matter more than GPR density.

Three dimensions of intentional extra detail distinguish EHMN 2026 from these comparators (Table S9.2). First, metabolite granularity: 11 compartments—including an explicit inner mitochondrial membrane (20 species for OXPHOS proton gradient reactions), peroxisome (844 species for very-long-chain fatty acid β-oxidation), and lysosome (640 species for degradative pathways)—and 855 chain-length-specific fatty acid and sphingolipid reactions representing C6–C26 variants as distinct species. This granularity is required for lipidomics data integration and lipotoxicity modelling; neither Human1 nor Recon3D currently represent chain-length variants as distinct reactions. Second, annotation architecture: a uniform MetaNetX MNXref namespace across 80.6% of metabolites, Reactome event IDs per reaction for 61% of the enzymatic core, and HGNC/ENSG gene encoding—all three simultaneously embedded in the deposited SBML. Third, thermodynamic completeness: 1923 reactions re-constrained based on biochemical evidence, 37 futile cycles eliminated, 0 remaining infeasible loops—a stricter standard than either comparator achieves.

GPR completeness is explicitly not a primary goal of EHMN 2026. The reconstruction prioritises stoichiometric completeness (retaining boundary and transport reactions required for flux solvability), thermodynamic plausibility (correct directionality for committed reactions), and annotation coherence (namespace-clean identifier layers). Speculative gene attribution—assigning GPR by homology inference without confirmed human biochemical evidence—would inflate the headline GPR percentage while reducing annotation precision. The 3996 HGNC-registered gene products in EHMN 2026, exceeding both Human1 (3628) and Recon3D (3288), demonstrate that breadth of gene coverage and conservative annotation philosophy are not in tension.

The GPR annotation philosophy in EHMN 2026 prioritises precision over completeness: associations are assigned only where a specific human gene can be confidently linked to the catalysed reaction. This contrasts with automated reconstruction pipelines (RAVEN, ModelSEED) that assign predicted GPR to most reactions, often including lower-confidence homology transfers. The resulting 62% core enzymatic coverage, applied to a larger enzymatic set than comparator models, offers higher specificity for gene knockout simulation, omics integration, and context-specific model extraction, where spurious GPR assignments would introduce false-positive predictions of gene essentiality.

### 4.3. Thermodynamic Coherence and Downstream Stability

Thermodynamic inconsistency remains a known vulnerability in constraint-based reconstructions. Infeasible energy-generating cycles can distort predictions, particularly when models are dynamically coupled to regulatory or pharmacological modules. The structured directionality refinement implemented in EHMN 2026 reduces such artefacts, improving feasibility without imposing full ΔG parameterisation. This refinement is especially important for integration into QSP contexts where metabolic states influence immune cell proliferation, cytokine secretion, or drug-target engagement [19,23]. Ensuring thermodynamic plausibility at the stoichiometric level strengthens model stability under multi-layer coupling. While a full genome-scale thermodynamic recalculation using the eQuilibrator component contribution framework was not performed in the present submission, structural and biochemical heuristics were applied to enforce plausible reaction directionality and eliminate energy-generating cycles. A comprehensive ΔG′–based quantitative refinement is planned as a subsequent extension. The current semi-quantitative approach differs from full parameterisation primarily in its reliance on discrete directionality constraints rather than continuous thermodynamic potentials. While the current heuristics—such as energy-currency cycle detection and biochemical irreversibility rules—successfully eliminate gross artefacts like unconstrained ATP-generating loops, a fully parameterised eQuilibrator-based model would provide more granular predictive accuracy. Specifically, full parameterisation allows for the calculation of ‘thermodynamic driving forces,’ which can identify near-equilibrium reactions that may shift directionality under varying physiological concentrations—a level of detail not fully captured by static stoichiometric bounds. Consequently, while EHMN 2026 offers high structural stability for standard flux balance analysis, future quantitative extensions will be required to capture the concentration-dependent metabolic flexibility essential for high-fidelity clinical simulations. The full ΔG′° extension will proceed in two stages. First, once MetaNetX harmonisation extends formula coverage to the ~3313 specific metabolites currently lacking annotation, the eQuilibrator component contribution method can be applied consistently across all reactions with annotated participants, eliminating the two-tier constraint inconsistency that makes partial application unsuitable. Second, intracellular metabolite concentration ranges from CYTOCON DB [19,20] will be incorporated to enable thermodynamic driving force analysis per flux distribution and identification of near-equilibrium reactions whose directionality shifts under varying physiological conditions. The current stoichiometric bounds will serve as the initial constraint skeleton for this quantitative layer, ensuring full backward compatibility with the current model version.

### 4.4. Pathway-Level Integration and Regulatory Extensibility

The Reactome annotation layer in EHMN 2026 provides a pathway-level organisational scaffold compatible with enrichment analysis and multi-omics integration workflows. The 61% metabolic-core coverage exceeds that of comparable human reconstructions and reflects active Reactome curation during the reconstruction pipeline. The remaining unannotated metabolic fraction arises from two bounded sources: 4730 legacy KEGG-lineage reactions (R*/RE* prefix) for which Reactome mapping is planned for the next release cycle, and 159 confirmed human biochemical reactions whose Reactome entries do not yet exist, representing a contribution pathway back to the source database.

Effective use of the Reactome annotation layer requires awareness of three coverage limitations that arise from Reactome’s curation scope rather than from the EHMN 2026 reconstruction. First, Reactome annotates transport processes at the pathway level but not individual transporter isoforms; the 1423 EHMN 2026 transport reactions therefore lack reaction-level Reactome IDs, and transporter gene analysis should use the gene-level mapping in Supplementary Data S4. Second, lipid chain-length variant reactions share a Reactome parent pathway ID but lack individual leaf event IDs; flux summation for fatty acid and sphingolipid pathways should aggregate at the parent level using the all-levels hierarchical annotation layer. Third, the 4730 legacy KEGG-lineage reactions (R*/RE* prefix) represent reaction classes rather than specific human biochemical events and are correctly absent from Reactome-based pathway analysis For the 12,969 MAR-curated enzymatic reactions—the biologically interpretable core of the reconstruction—Reactome coverage is 61%, the highest reported for any current human GEM, and the leaf-level annotation layer provides a clean non-redundant input to standard pathway enrichment tools. See Supplementary S7.

Specifically, regulatory databases such as TRANSFAC [28,29] and signalling knowledgebases such as TRANSPATH [30,31] provide structured representations of transcription factor control and signal transduction cascades. Converting these resources into SBML-compatible modules would allow dynamic modulation of metabolic gene expression and enzyme activity within the EHMN framework.

Such multi-layer coupling would establish a signalling → transcription → metabolism cascade capable of representing drug-induced or inflammation-driven metabolic rewiring.

### 4.5. Bridging Genome-Scale Metabolism and QSP

Quantitative systems pharmacology increasingly integrates mechanistic disease biology with clinical pharmacokinetics and pharmacodynamics. Databases such as CYTOCON and CYTOCON DB provide curated in vivo baseline concentrations of human immune cells and mediators, supporting calibration of QSP models [19,20].

Embedding EHMN 2026 within such QSP frameworks enables:

- Metabolic flux states influencing immune activation dynamics;
- Cytokine signalling constraining metabolic pathway utilisation;
- Drug perturbations propagating from receptor engagement to metabolic adaptation.

Hybrid QSP approaches combining mechanistic modelling and machine learning are rapidly emerging [22,23,24], and genome-scale metabolic structure provides a critical mechanistic constraint layer for such AI-augmented systems.

### 4.6. Selective Kinetic Augmentation via Curated Databases

While full kinetic conversion of genome-scale networks remains infeasible, selective kinetic augmentation offers a tractable strategy.

SABIO-RK provides curated reaction-level kinetic laws and parameters suitable for SBML embedding [32], while BRENDA supplies enzyme-centric kinetic constants and functional annotations [33]. These resources enable tiered kinetic enrichment at key metabolic control points, drug targets, or disease-relevant pathways.

A selective kinetic uplift strategy preserves the scalability of the stoichiometric backbone while introducing dynamic behaviour where biologically necessary. This approach aligns with QSP best practices emphasising modularity and parsimony [21,24].

The comparatively lower GPR coverage relative to Recon3D and Human1 should not be interpreted as reduced annotation quality. Instead, it reflects a reconstruction philosophy that prioritises stoichiometric completeness and thermodynamic plausibility while avoiding speculative gene assignment. Many reactions retained in EHMN 2026 represent transport processes, exchange reactions, or balancing transformations that lack direct gene annotation but are necessary for network closure.

In practical terms: standard FBA operates on all 22,642 reactions and is unaffected by GPR density; gene knockout screening and transcriptomic/proteomic integration operate on the 9638 GPR-annotated reactions (62% of enzymatic core) and are fully supported for all major metabolic pathways; context-specific model extraction using FASTCORE, iMAT, or tINIT applies GPR constraints to annotated reactions and treats Categories A and D reactions as always-active, consistent with standard practice in Human1 and Recon3D; and QSP/multi-omics integration is governed by identifier coherence rather than GPR density, where the MetaNetX-harmonised namespace is the primary enabler. Quantitative systems pharmacology increasingly integrates mechanistic disease biology with pharmacokinetics and pharmacodynamics [33].

### 4.7. Toward AI-Assisted Continuous Model Evolution

As biomedical knowledge expands rapidly, static reconstructions risk obsolescence. Semi-automated updating pipelines leveraging literature extraction, identifier harmonisation, and regression validation can maintain model relevance.

Because EHMN 2026 is fully standards-compliant and annotation-dense, it is structurally amenable to AI-assisted updating workflows. Such pipelines could integrate new kinetic parameters (from SABIO-RK or BRENDA), regulatory relationships (TRANSFAC), signalling cascades (TRANSPATH) [28,29,30,31,34], or immune baseline data (CYTOCON) into a version-controlled SBML ecosystem [19,35].

This evolutionary architecture shifts genome-scale reconstruction from a static artefact toward a continuously curated modelling infrastructure.

The upgraded EHMN reconstruction provides a flexible computational framework that supports multiple types of systems biology analyses. First, the chemically consistent and standardised network structure enables its direct use in constraint-based metabolic modelling approaches, including flux balance analysis and related optimisation methods. Second, the harmonised gene–protein–reaction associations allow the model to be integrated with transcriptomic, proteomic and metabolomic datasets, enabling data-driven analysis of metabolic states across tissues, diseases, or experimental conditions. Finally, the improved annotation and interoperability of the reconstruction facilitate its incorporation into (QSP) and wider MIDD models, where metabolic pathways can be coupled with signalling, regulatory, and pharmacokinetic/pharmacodynamic processes to study drug response and disease mechanisms at a systems level.

### 4.8. Limitations

EHMN 2026 remains primarily stoichiometric, and kinetic embedding is not globally implemented. Thermodynamic refinement successfully addresses global feasibility and eliminates infeasible energy-generating cycles; however, it does not yet incorporate full Gibbs free-energy parameterisation. This reliance on semi-quantitative heuristics rather than continuous thermodynamic potentials means the model may not yet capture the concentration-dependent directionality shifts in near-equilibrium reactions. Additionally, systematic benchmarking across multi-cohort metabolomics and clinical QSP case studies remains an important future step. Nevertheless, the reconstruction’s emphasis on coherence, interoperability, and modular extensibility positions it as a stable foundation for translational and systems pharmacology integration.

## 5. Conclusions

EHMN 2026 advances human metabolic reconstruction beyond reaction expansion toward structurally engineered integrability. By combining thermodynamic coherence, identifier harmonisation, pathway hierarchy integration, and SBML Level 3 FBC compliance, it provides a metabolic backbone optimised for integration into QSP, regulatory, and kinetic modelling frameworks.

Through future coupling with CYTOCON [19,20], kinetic repositories such as SABIO-RK and BRENDA [32,33,34], and regulatory databases including TRANSFAC and TRANSPATH [28,29,30,31], EHMN 2026 can evolve into a multi-layer, dynamically extensible systems biology platform.

Mechanistic AI and QSP integration approaches are increasingly used to couple metabolic networks with drug discovery models [36].

## Figures and Tables

**Table 1:** The GPR coverage difference (42.6% for EHMN 2026 vs. ~96–97% for Human1/Recon3D) reflects reconstruction philosophy rather than annotation quality: EHMN 2026 retains a larger boundary reaction set (exchange + sink/demand: 6476 reactions, 28.6% of total) that is necessary for stoichiometric solvability but carries no enzymatic gene. Restricting to the enzymatic MAR core yields 62.0% coverage; see Section GPR Coverage: Reconstruction Philosophy and Downstream Implications.

Metric	EHMN 2026	Human1	Recon	EHMN vs. Avg	Reconstruction Philosophy
Reactions	22,642	13,416	13,543	+68%	Annotation-dense foundation model for QSP integration (EHMN)/Global consensus GPR coverage (Human1, Recon3D)
Metabolites	14,321	8378	4140	+91%	14,321 species across 11 compartments (EHMN)/Consensus metabolite sets (Human1, Recon3D)
Compartments	11	13	10	-	11 compartments enabling subcellular pathway separation (EHMN)/13 and 10 compartments (Human1, Recon3D)
Gene products	3996	3628	3288	+10%	Isoform-resolved gene products with ENSG standardisation (EHMN)/Near-complete gene coverage (Human1, Recon3D)
Exchange reactions	248	~622	~469	Constrained	Boundary conditions modelled explicitly (EHMN)/Flux-unconstrained by default (others)
Transport reactions	1423	~1000	~900	+43–58%	Transport isoforms retained for compartmental detail (EHMN)
Sink reactions	3114	—	—	Novel	Structural boundary conditions modelled; not present in Recon3D/Human1
GPR coverage (all rxns)	42.6%	~96%	~97%	-	GPR density reflects reconstruction scope, not quality (EHMN)/Automated completion approach (Human1, Recon3D)
GPR coverage (enzymatic core)	62.0%	~96%	~97%	-	62% over enzymatic core—like-for-like with Human1/Recon3D (EHMN)
Irreversible rxns	43.2%	~40%	~35%	+3–8 pp	Thermodynamic directionality refinement applied (EHMN)/Heuristic bounds (others)
Blocked reactions	227	~200	~150	-	Post-deduplication; comparable rates expected in others
Stoichiometric non-zeros	104,469	—	—	-	High stoichiometric density enabling precise QSP embedding (EHMN)
Unique Reactome IDs	2194	—	—	Novel	Native SBML Reactome annotation for pathway-level flux queries (EHMN only)
ChEBI coverage	53.6%	Partial	—	-	Explicit ChEBI reconciliation for thermodynamic compatibility (EHMN)/Partial (Human1)/Not reported (Recon3D)

**Table 2:** No-GPR Reaction Class Breakdown.

Cat.	Reaction Class	Count	% of 13,004 No-GPR	Why GPR Is Not Expected or Not yet Assigned	Impact on Downstream Analyses	Future Coverage?
A	Exchange & sink/demand boundary reactions	3362 + 3114 = 6476	49.8%	Structural boundary conditions representing environmental flux constraints; no enzyme catalyses these by definition.	None—excluded from all GPR-dependent analyses by design. Context-specific model extraction (FASTCORE, iMAT) ignores boundary reactions.	No
B	Transport isoforms without confirmed HGNC transporter gene	1063	8.2%	Many transport mechanisms are mediated by poorly characterised or non-specific transporters; HGNC gene not yet confirmed in the literature.	Reduced: transporter gene expression data cannot be integrated for these reactions; they remain active/inactive based on flux bounds only. Does not affect central metabolic pathway simulations.	Partial—as SLC/ABC transporter annotation improves.
C	Legacy KEGG/BRENDA-lineage reactions (R*, RE* prefixes)	4730	27.0%	Reactions from KEGG source reconstruction not yet systematically mapped to human gene assignments in this release cycle.	Reduced for KEGG-lineage pathways: transcriptomic integration limited for R*/RE* reactions. Mapped in MAR lineage where available. Planned for next release.	Yes—planned for next release.
D	FA/sphingolipid chain-length variant reactions	855	6.6%	Gene is assigned to the canonical chain-length form only; chain-length-variant reactions share the same enzyme but are represented as distinct reactions for stoichiometric granularity.	Minimal: the canonical-form GPR still permits gene knockout simulation for the enzyme class; chain-length specificity is for metabolite granularity not gene coverage.	Partial—canonical GPR covers the gene.
E	MAR reactions where no human gene can be confidently assigned	4072	31.3%	Includes: 1478 unnamed source-database entries; reactions that are spontaneous or non-enzymatic in human physiology; reactions from non-human pathways retained for network scope.	Targeted: reactions in this category cannot be used for gene knockout or transcriptomic integration but remain valid for stoichiometric flux analysis. 36–86% subsystem-level saturation reflects experimental characterisation gradient.	Partial—spontaneous/non-human subset will not gain GPR; unnamed entries are future targets.

Category E subsystem-level GPR saturation ranges from 36% (xenobiotic/drug metabolism) to 86% (vitamins and cofactors), consistent with differential experimental characterisation in the biochemical literature. Categories A–E are non-exclusive: a reaction may belong to more than one class (e.g., a KEGG-lineage exchange reaction belongs to both Cat A and Cat C). The sum of category counts (15,980) therefore exceeds 13,004, and column percentages sum to 122.9%. * Reactions with prefix R* or RE* originate from KEGG-lineage source databases and have not yet been mapped to confirmed human gene assignments in this release cycle.

**Table 3:** Downstream Analysis Impact by Analysis Type.

Analysis Type	Reactions Usable	Effective Coverage	Recommended Denominator/Note
Standard FBA/flux balance analysis	All 22,642	Full: GPR associations not required for stoichiometric simulation.	Use all reactions. GPR coverage irrelevant for FBA per se.
Gene knockout/essentiality screening	9638 GPR-annotated reactions	62% of MAR enzymatic core (8042/12,969). All 3996 gene products screened.	Use MAR-core denominator. Cat. A reaction correctly excluded. Cat. C/E reactions retained as unaffected by knockout.
Transcriptomic integration (FASTCORE, iMAT, tINIT)	9638 GPR-annotated reactions	62% of enzymatic core. Expression thresholds applied to GPR-annotated reactions; remainder treated as always-active or flux-bound constrained.	Standard practice: boundary/transport reactions without GPR are treated as unconstrained or always active. This is identical to how Human1 and Recon3D handle their own non-enzymatic reactions.
Context-specific model extraction (tissue/cell-type)	9638 GPR-annotated reactions	Same as transcriptomic integration. 62% enzymatic-core coverage sufficient for context extraction of central carbon, amino acid, lipid, and OXPHOS pathways.	Cat. A/B reactions are typically included in extracted models as flux-bound active. Cat. C legacy reactions are excluded from expression-guided extraction unless manually curated.
Proteomics integration	9638 GPR-annotated reactions	62% enzymatic core. Protein abundance maps to enzyme capacity via GPR rules.	GECKO-style enzyme capacity constraints apply to GPR-annotated reactions. Protein pool constraint already implemented in EHMN 2026 for MAR reactions.
Drug target/pathway impact analysis	9638 GPR-annotated reactions + Reactome pathway IDs on 7910 reactions	Drug target gene → GPR → reaction → pathway. Reactome IDs on reaction enable on/off-target metabolic impact without external mapping.	One of the most comprehensive analysis frameworks among current human GEMs: 3996 genes × Reactome IDs simultaneously available per reaction.
Multi-omics/QSP integration	All GPR-annotated reactions + Reactome layer	Clean HGNC/ENSG namespace enables direct mapping to transcriptomics, proteomics, and pharmacological datasets without ID translation.	Uniform MetaNetX namespace is the key advantage here; 42.6% headline figure does not constrain QSP coupling, which operates on flux states rather than per-reaction GPR.

**Table 4:** Thermodynamic irreversibility by metabolic pathway class in EHMN 2026.

Pathway/Class	N Reactions	Irreversible (%)	Interpretation
OXPHOS/ETC	25	80.0%	Thermodynamically committed; consistent with proton-motive-force directionality
Fatty Acid β-oxidation	303	59.1%	Majority irreversible; ~8 NADH + 2 FADH2 per cycle constrained forward
Amino Acid Metabolism	41	51.2%	Near-equal split reflects reversible transamination equilibria
TCA Cycle reactions	1	0.0%	Detected fragment; full TCA coverage in MAR subset below
Transport (RT)	1423	34.2%	Predominantly reversible; 132 blocked (tissue-specific gates)
Exchange (EX)	248	0.0%	All bidirectional by design; uptake/secretion unconstrained
Metabolic (MAR)	12,969	54.0%	Core enzymatic reactions; 65 blocked after thermo curation

Footnote. GPR coverage of 42.6% is reported over all 22,642 reactions including 3362 exchange, 3114 sink/demand, and 1423 transport reactions that carry no enzymatic gene by design. Coverage of the 12,969 MAR-curated enzymatic reactions is 62.0%, computed on a core enzymatic set larger than either Human1 or Recon3D. EHMN 2026 encodes 3996 unique HGNC genes, exceeding both comparators. GPR-free reactions comprise five categories: boundary/sink reactions (26%), legacy KEGG reactions awaiting curation (27%), transport isoforms without a confirmed gene (8%), lipid chain-length expansion variants (7%), and MAR reactions with incomplete or absent human gene assignment (31%); none represent enzymatic reactions in which the catalysing human gene is known but unrecorded. Counts and percentages were computed from the deposited EHMN 2026 SBML file after thermodynamic curation.

**Table 5:** Semi-quantitative vs. Full ΔG′° Parameterisation.

Criterion	EHMN 2026 (Current Submission)	Full eQuilibrator ΔG′° Parameterisation (Planned)
Method	Biochemical irreversibility rules (ATP ligases, decarboxylations, NADH/NADPH reductions with ΔE° > +100 mV, OXPHOS/ETC, β-oxidation); iterative boundary-closure LP for cycle detection. A total of 1923 reactions re-constrained; 37 cycles resolved.	Component contribution method applied to all reactions with complete metabolite formula + charge; continuous ΔG′° per reaction at pH 7.4, T = 310 K.
What it achieves	Eliminates all ATP-generating futile cycles (0 remaining verified in deposited SBML). Correct directionality for all reactions where |ΔG′°| ≫ 0 and biochemical basis is unambiguous. Stable FBA solution space.	All of the above PLUS: identification of near-equilibrium reactions whose directionality shifts with metabolite concentrations; thermodynamic driving force calculation per flux distribution.
What it cannot capture	Near-equilibrium reactions (|ΔG′°| < ~10–15 kJ/mol) where physiological metabolite concentrations determine directionality—e.g., lactate dehydrogenase, malate dehydrogenase, some aminotransferases.	Reactions without complete metabolite formula annotation (~38.8% of EHMN 2026 species currently); computationally intensive; requires intracellular concentration data.
Why this approach for current submission	(1) 38.8% species lack formulas—applying ΔG′° to ~61% of reactions and heuristics to 39% creates internally inconsistent two-tier constraints. (2) No published human GEM has applied full eQuilibrator at genome scale—EHMN 2026 is consistent with and exceeds community practice. (3) Conservative: retains reversibility for near-equilibrium reactions where forced directionality would be speculative.	Prerequisite: extend formula coverage to ~3313 remaining specific metabolites; incorporate metabolite concentration ranges from CYTOCON DB. Planned for next release cycle.
Literature comparators	Recon3D: heuristic; ~35% irreversible; no published cycle-resolution pipeline. Human1 [7]: heuristic; ~40% irreversible. iDopaNeuro [6,7]: heuristic + manual curation. None applies full eQuilibrator at genome scale.	Not yet published for human GEMs.
Internal validation evidence	OXPHOS/ETC 80% irreversible; β-oxidation 59.1%; amino acid metabolism 51.2%; exchange 0%—hierarchy matches biochemical expectations (Table 2, manuscript). In total, 0 unconstrained cycles in deposited SBML.	N/A—future work.

**Table 6:** GPR Coverage.

Denominator Definition	GPR-Annotated/Total	Coverage
All 22,642 reactions (headline figure)	9638/22,642	42.6%
Excl. exchange + sink/demand reactions	9638/19,280	50.0%
Excl. exchange + sink + transport	9274/17,853	51.9%
Excl. exchange + sink + transport + legacy (R*/RE*)	8058/13,123	61.4%
MAR curated enzymatic reactions only	8042/12,969	62.0%

62% GPR coverage on 12,969 MAR enzymatic reactions—a denominator ~18% larger than the complete enzymatic sets of either Human1 (~10,900) or Recon3D (~11,000). EHMN 2026’s 3996 unique HGNC-registered genes already exceed both comparators (Human1: 3628; Recon3D: 3288). The lower overall GPR percentage relative to Human1 and Recon3D therefore reflects two features that are architectural strengths, not deficits: (i) a larger boundary reaction set for simulation completeness, and (ii) a conservative GPR assignment philosophy that avoids speculative gene attribution. The 62% enzymatic-core figure is the appropriate metric for comparing annotation quality. All counts were computed directly from the deposited SBML model and reaction-class partitioning described in Section GPR Coverage: Reconstruction Philosophy and Downstream Implications. * Reactions with prefix R* or RE* are KEGG-lineage reactions not yet assigned to confirmed human genes in this release cycle.

**Table 7:** Architectural Feature Comparison.

Feature	EHMN 2026	Recon3D [6]	Human1 [7]
Metabolite identifier harmonisation	80.6% MetaNetX/MNXref; 53.6% ChEBI-validated; uniform namespace across all 14,321 species	Partial; heterogeneous legacy IDs (KEGG/BiGG/HMDB); no systematic MNXref reconciliation reported	Partial; BiGG/KEGG hybrid namespace; cross-model reconciliation requires external mapping
Reactome pathway annotation (reaction-level, embedded in SBML)	7910 reactions annotated; 2194 unique Reactome IDs embedded in SBML annotation blocks; 61% of MAR enzymatic core	Not natively included; third-party overlays yield ~6–15% reaction coverage; no SBML-embedded IDs	Subsystem labels present; no native Reactome event IDs in SBML annotation blocks
Thermodynamic cycle resolution (documented, reproducible)	37 infeasible cycles identified and fully resolved; 9792 reactions set irreversible (43.2%); 0 remaining loops verified in deposited SBML	~35% irreversible; no published iterative cycle-detection pipeline; some infeasible cycles documented in the literature	~40% irreversible; partial directionality curation; no published full cycle-elimination pass
SBML Level 3 + FBC2 compliance	Full compliance; explicit geneProductAssociation encoding; libSBML-validated; no orphan references; zero embedded kinetic laws	SBML compliant; predates FBC2 geneProducts structure; some analysis tools require conversion step	SBML compliant; geneProduct encoding present; some legacy Entrez IDs remain alongside HGNC
Metabolite granularity & compartment architecture	14,321 species across 11 compartments; explicit inner mitochondrial space (20 species), lysosome (640), peroxisome (844); FA chain-length variants represented as distinct reactions	5835 species; 10 compartments; lipid classes aggregated; no inner mitochondrial separation	8378 species; 13 compartments (incl. sub-regions); some lipid granularity; no explicit inner mito space
Gene product count & identifier standard	3996 HGNC symbols; 2887 unique ENSG IDs; exceeds both comparators; compatible with current Ensembl/UniProt pipelines	3288 HGNC symbols	3628 HGNC symbols; some legacy Entrez IDs remain
GPR coverage—enzymatic core (fair comparison denominator)	62% (8042/12,969 MAR reactions); conservative philosophy—no speculative homology transfers	~97% (all reactions); automated pipeline includes predicted GPR via homology	~96% (all reactions); RAVEN/HMM-based GPR; near-complete but includes predicted assignments
QSP/multi-layer integration readiness	Designed as QSP substrate; uniform namespace prevents ID conflicts in coupled PBPK/PD modules; Reactome layer bridges to signalling databases (TRANSFAC, TRANSPATH)	Primary use: FBA; not designed for QSP; no pathway annotation layer; namespace issues in coupled models	Primary use: FBA and context-specific extraction; not designed for QSP coupling; no Reactome layer

Third-party Reactome coverage estimates for Recon3D and Human1 from PathwayCommons/Enrichr overlays; neither model includes native Reactome event IDs in SBML annotation blocks.

**Table 8:** Modelling Tasks and Architectural Fit.

Modelling Task	Why EHMN 2026 Architecture Matters	Best-Suited Model
Standard FBA/flux balance analysis	All three models are viable. EHMN 2026’s higher irreversibility (43.2% vs. ~35–40%) yields a more constrained solution space; OXPHOS at 80% and β-oxidation at 59% irreversibility reduce artefactual reverse-flux predictions.	All three suitable
Gene essentiality/knockout screening	EHMN 2026 provides the highest confirmed gene count (3996). The conservative GPR philosophy avoids false-positive essentiality calls: associations are assigned only where a specific human gene is confirmed, not via homology prediction.	EHMN 2026 preferred
Transcriptomics/proteomics integration	HGNC-standardised gene IDs map directly to RNA-seq and proteomics datasets. 62% GPR on the 12,969-reaction MAR core supports FASTCORE, tINIT, or iMAT extraction. Uniform MetaNetX namespace reduces ID-translation overhead.	EHMN 2026 or Human1
Pathway-level flux aggregation (subsystem summaries)	EHMN 2026 is the only model with native Reactome event IDs embedded per reaction. This allows direct pathway-level flux summation (e.g., all “Cholesterol biosynthesis” reactions) and hierarchical aggregation without external mapping tables.	EHMN 2026 uniquely
Organelle-specific perturbation (lysosomal storage, peroxisomal defects, Complex I inhibition)	Eleven-compartment architecture with explicit inner mitochondrial space (20 species), lysosome (640 species), and peroxisome (844 species) allows organelle-level flux isolation not feasible in 8–10 compartment models.	EHMN 2026 uniquely
QSP/PBPK + signalling + metabolism coupling	Uniform MetaNetX namespace eliminates metabolite ID conflicts across model layers. Thermodynamic consistency prevents infeasible flux states from propagating instability into regulatory/pharmacological modules. Reactome IDs provide structural links to signalling and regulatory knowledgebases such as TRANSFAC and TRANSPATH [28,29,30,31].	EHMN 2026 uniquely
Drug–target metabolic impact (pathway-level)	Gene → Reactome pathway IDs embedded per reaction allow intersection with ChEMBL/DrugBank target databases without external table joins. Combined 3996 gene products support on-target and off-target metabolic pathway analysis.	EHMN 2026 preferred
Lipid subclass/FA chain-length specific modelling	Chain-length variants are represented as distinct reactions (a significant share of the 12,969 MAR reactions). Enables LCAD/MCAD/SCAD deficiency modelling, sphingolipid signalling, and β-oxidation efficiency studies that aggregate-class models cannot resolve.	EHMN 2026 preferred

## Data Availability

The final EHMN 2026 genome-scale reconstruction is encoded in SBML Level 3 Version 2 with Flux Balance Constraints (FBC) extension and will be publicly deposited prior to publication. The model will be available through: IQANOVA Repository A mirrored version of the SBML model, associated Supplementary Materials (S1–S4), and version-controlled update history will be hosted at: http://www.iqanova.org (accessed 30 March 2026) The SBML file is available on request from the corresponding author. Version Control and Traceability Each released model version will include: Unique version identifier (EHMN_2026_v1.0) SHA256 checksum of SBML file Change log documenting structural, thermodynamic, or annotation updates Cross-reference compatibility with BioModels accession Reproducibility Notes The model is fully compatible with: COBRA Toolbox v3.0 COBRApy SBML-compliant solvers Tellurium/libRoadRunner All refinement workflows described in Section 2.2 and Section 2.3 are reproducible using the supplementary mapping and directionality tables provided.

## Supplementary Materials

The following supporting information can be downloaded at: https://www.mdpi.com/article/10.3390/metabo16040236/s1.

## References

[1] Orth J.D., Thiele I., Palsson B.Ø. (2010). What is flux balance analysis?. Nat. Biotechnol..

[2] Bordbar A., Monk J.M., King Z.A., Palsson B.Ø. (2014). Constraint-based models predict metabolic and associated cellular functions. Nat. Rev. Genet..

[3] Lewis N.E., Nagarajan H., Palsson B.Ø. (2012). Constraining the metabolic genotype–phenotype relationship using a phylogeny of in silico methods. Nat. Rev. Microbiol..

[4] Varma A., Palsson B.Ø. (1994). Metabolic flux balancing: Basic concepts, scientific and practical use. Nat. Biotechnol..

[5] Thiele I., Swainston N., Fleming R.M.T., Hoppe A., Sahoo S., Aurich M.K., Haraldsdottir H., Mo M.L., Rolfsson O., Stobbe M.D. (2013). A community-driven global reconstruction of human metabolism. Nat. Biotechnol..

[6] Brunk E., Sahoo S., Zielinski D.C., Altunkaya A., Dräger A., Mih N., Gatto F., Nilsson A., Preciat Gonzalez G.A., Aurich M.K. (2018). Recon3D enables a three-dimensional view of gene variation in human metabolism. Nat. Biotechnol..

[7] Cervettini D., Tang S., Fried S.D., Willis J.C.W., Funke L.F.H., Colwell L.J., Chin J.W. (2020). An atlas of human metabolism. Nat. Biotechnol..

[8] Moretti S., Martin O., Van Du Tran T., Bridge A., Morgat A., Pagni M. (2016). MetaNetX/MNXref: Reconciliation of metabolites and biochemical reactions to bring together genome-scale metabolic networks. Nucleic Acids Res..

[9] Hastings J., Owen G., Dekker A., Ennis M., Kale N., Muthukrishnan V., Turner S., Swainston N., Mendes P., Steinbeck C. (2016). ChEBI in 2016: Improved services and an expanding collection of metabolites. Nucleic Acids Res..

[10] Beard D.A., Qian H. (2005). Thermodynamic-based computational profiling of cellular regulatory control in hepatocyte metabolism. Am. J. Physiol. Endocrinol. Metab..

[11] Henry C.S., Broadbelt L.J., Hatzimanikatis V. (2007). Thermodynamics-based metabolic flux analysis. Biophys. J..

[12] Noor E., Bar-Even A., Flamholz A., Reznik E., Liebermeister W., Milo R. (2014). Pathway thermodynamics highlights kinetic obstacles in central metabolism. PLoS Comput. Biol..

[13] Ma H., Sorokin A., Mazein A., Selkov A., Selkov E., Demin O., Goryanin I. (2007). The Edinburgh human metabolic network reconstruction and its functional analysis. Mol. Syst. Biol..

[14] Fabregat A., Jupe S., Matthews L., Sidiropoulos K., Gillespie M., Garapati P., Haw R., Jassal B., Korninger F., May B. (2022). The Reactome pathway knowledgebase 2022. Nucleic Acids Res..

[15] Hucka M., Bergmann F.T., Chaouiya C., Dräger A., Hoops S., Keating S.M., König M., Le Novère N., Myers C.J., Olivier B.G. (2019). The Systems Biology Markup Language (SBML): Language specification for Level 3 Version 2 Core. J. Integr. Bioinform..

[16] Olivier B.G., Bergmann F.T. (2018). SBML Level 3 Package: Flux Balance Constraints version 2. J. Integr. Bioinform..

[17] Huh Y., Riley S., Nicholas T. (2020). Quantitative systems pharmacology modeling of metabolism and disease. CPT Pharmacomet. Syst. Pharmacol..

[18] Robinson J.L., Nielsen J. (2016). Integrative analysis of human omics data using biomolecular networks. Mol. Biosyst..

[19] Leonov V., Mogilevskaya E., Gerasimuk E., Gizzatkulov N., Demin O. (2023). CYTOCON: The manually curated database of human in vivo cell and molecule concentrations. CPT Pharmacomet. Syst. Pharmacol..

[20] Desikan R., Jayachandran P. (2023). CYTOCON DB: A versatile database of human cell and molecule concentrations for accelerating model development. CPT Pharmacomet. Syst. Pharmacol..

[21] Elmokadem A., Riggs M.M., Baron K.T. (2019). Quantitative Systems Pharmacology and PBPK Modeling with Mrgsolve: A Hands-On Tutorial. CPT Pharmacomet. Syst. Pharmacol..

[22] Aghamiri S.S., Amin R., Helikar T. (2022). Recent applications of QSP and machine learning models across diseases. J. Pharmacokinet. Pharmacodyn..

[23] Cheng L., Qiu Y., Schmidt B.J., Wei G.-W. (2022). Applications of quantitative systems pharmacology modeling and machine learning for heart failure. J. Pharmacokinet. Pharmacodyn..

[24] Lemaire V., Hu C., van der Graaf P.H., Chang S., Wang W. (2024). No recipe for quantitative systems pharmacology model development. Clin. Pharmacol. Ther..

[25] Lieven C., Beber M.E., Olivier B.G., Bergmann F.T., Ataman M., Babaei P., Bartell J.A., Blank L.M., Chauhan S., Correia K. (2020). MEMOTE for standardized genome-scale metabolic model testing. Nat. Biotechnol..

[26] Keating S.M., Waltemath D., König M., Zhang F., Dräger A., Chaouiya C., Bergmann F.T., Finney A., Gillespie C.S., Helikar T. (2020). SBML Level 3: An extensible format for the exchange and reuse of biological models. Mol. Syst. Biol..

[27] Goryanin I., Goryanin I., Demin O. (2025). Revolutionizing drug discovery: Integrating artificial intelligence with quantitative systems pharmacology. Drug Discov. Today.

[28] Wingender E., Chen X., Hehl R., Karas H., Liebich I., Matys V., Meinhardt T., Prüss M., Reuter I., Schacherer F. (2000). TRANSFAC: An integrated system for gene expression regulation. Nucleic Acids Res..

[29] Matys V., Fricke E., Geffers R., Gößling E., Haubrock M., Hehl R., Hornischer K., Karas D., Kel A.E., Kel-Margoulis O.V. (2003). TRANSFAC: Transcriptional regulation from patterns to profiles. Nucleic Acids Res..

[30] Schacherer F., Choi C., Götze U., Krull M., Pistor S., Wingender E. (2001). The TRANSPATH signal transduction database. Bioinformatics.

[31] Krull M., Voss N., Choi C., Pistor S., Potapov A., Wingender E. (2003). TRANSPATH: Integrated database on signal transduction and a tool for array analysis. Nucleic Acids Res..

[32] Wittig U., Rey M., Weidemann A., Kania R., Müller W. (2018). SABIO-RK: An updated resource for manually curated biochemical reaction kinetics. Nucleic Acids Res..

[33] Chang A., Jeske L., Ulbrich S., Hofmann J., Koblitz J., Schomburg I., Neumann-Schaal M., Jahn D., Schomburg D. (2021). BRENDA, the ELIXIR core data resource in 2021. Nucleic Acids Res..

[34] Wittig U., Kania R., Golebiewski M., Rey M., Shi L., Jong L., Algaa E., Weidemann A., Sauer-Danzwith H., Mir S. (2011). SABIO-RK database for biochemical reaction kinetics. Nucleic Acids Res..

[35] Sorger P.K., Allerheiligen S.R., Abernethy D.R., Altman R.B., Brouwer K.L., Califano A., D’Argenio D.Z., Iyengar R., Jusko W.J., Lalonde R. (2011). Quantitative and systems pharmacology in the post-genomic era: New approaches to discovering drugs and understanding therapeutic mechanisms. An NIH White Paper by the QSP Workshop Group.

[36] Schellenberger J., Lewis N.E., Palsson B.Ø. (2011). Elimination of thermodynamically infeasible loops in steady-state metabolic models. Biophys. J..

