# Supplementary material for: EHMN 2026: A Thermodynamically Refined, SBML-Standardised Human Metabolic Network for Genome-Scale Analysis and QSP Integration
Source: Metabolites. 2026 Mar 31;16(4):236. doi: 10.3390/metabo16040236 (PMC13118034; doi:10.3390/metabo16040236)
Supplement: Supplementary file 1 [file metabolites-16-00236-s001.zip › Supplementary S7.pdf]

Supplementary S7

— Using Reactome Annotations in EHMN 2026: Practical Guide

EHMN 2026 provides Reactome pathway annotations at two complementary levels: a full hierarchical layer (all Reactome event IDs at every level of the pathway hierarchy) and a leaf-level layer (terminal event IDs only, with hierarchical duplication removed). The two layers serve different analytical purposes and should not be interchanged (Table 8).

Hierarchical annotation is appropriate for broad biological categorisation, subsystem-level enrichment analysis, and heat-map visualisation of pathway-level flux distributions. Because parent pathway IDs are retained alongside child event IDs, a single reaction may carry 3–15 Reactome identifiers. This provides navigational context but inflates coverage statistics if used for quantitative summation.

Leaf-level annotation is the correct layer for quantitative pathway-level flux summation, perturbation analysis at specific event granularity, and input to standard pathway analysis tools (ReactomePA, g:Profiler) that expect non-redundant reaction or gene lists. At this level, each annotated reaction carries 1–3 event IDs, and the 1,278 reactions assigned to ≥1 leaf pathway (642 unique leaf events) can be aggregated without double-counting.

Three practical examples are provided: (1) pathway-level flux summation using the leaf layer to compute total flux through the cholesterol biosynthesis pathway (R-HSA-191273) under glucose constraint; (2) gene knockout perturbation analysis using single_gene_deletion to identify which leaf pathways show the greatest flux reduction after HMGCR knockout; and (3) Reactome gene-set enrichment from a context-specific model extraction output, using the HGNC gene-to-Reactome mapping in Supplementary Data S4 as input to the ReactomePA Bioconductor package. COBRApy and R code for all three examples are provided in Supplementary Data S5.

Users should be aware of three classes of reactions that are not covered by Reactome annotation and should be excluded from pathway-level analysis (Table 9). Transport reactions (1,427) are annotated at the process level in Reactome but not as individual isoform events; the gene-level mapping in Supplementary Data S4 should be used for transporter gene analysis. Fatty acid and sphingolipid chain-length variants (855) share a Reactome parent pathway ID but lack individual leaf event IDs; flux summation for these pathways should use all-levels annotation aggregated at the parent level. Legacy KEGG-lineage reactions (3,514, R*/RE* prefix) represent reaction classes rather than specific human biochemical events and are correctly absent from Reactome-based pathway analysis. Exchange and sink/demand boundary reactions (6,476) should always be excluded from pathway queries by filtering on the EX_*, SINK_*, and DM_* reaction ID prefixes.

Worked Examples (COBRApy)

These three examples are proposed for inclusion in the new Section 3.3.1 of the manuscript. They illustrate the three use cases the reviewer specified: pathway flux summation, perturbation analysis, and enrichment-ready output.

Example 1 — Pathway-level flux summation (leaf-level layer)

Scenario: sum total flux through the "Cholesterol biosynthesis" Reactome pathway under a standard glucose uptake constraint.

> **When to use this: quantifying total pathway throughput; comparing pathway activity between conditions; identifying rate-limiting steps within a defined Reactome event.**
>
> Use the leaf-level annotation layer. Aggregating at parent pathway level with hierarchical IDs would double-count reactions that belong to both "Cholesterol biosynthesis" and "Lipid metabolism" parent nodes.

```
import cobra
import json

# Load EHMN 2026
model =
cobra.io.read_sbml_model("EHMN_2026_repaired_pipeline_SBML_L3V2.xml")

# Load leaf-level Reactome mapping (Supplementary Data S4)
# Format: {reaction_id: [leaf_pathway_id1, leaf_pathway_id2, ...]}
with open("ehmn2026_leaf_reactome_map.json") as f:
    leaf_map = json.load(f)

# Target pathway: Cholesterol biosynthesis (Reactome R-HSA-191273)
TARGET_PATHWAY = "R-HSA-191273"

# Get reactions in target pathway
pathway_rxns = [r for r, ids in leaf_map.items() if TARGET_PATHWAY in ids]
print(f"Reactions in {TARGET_PATHWAY}: {len(pathway_rxns)}")

# Run FBA
with model:
    model.reactions.get_by_id("EX_glc__D_e").lower_bound = -10  # glucose
uptake
    sol = model.optimize()

# Sum absolute flux through pathway reactions
if sol.status == "optimal":
    pathway_flux = sum(abs(sol.fluxes[r]) for r in pathway_rxns
                       if r in sol.fluxes)
    print(f"Total cholesterol biosynthesis flux: {pathway_flux:.4f}
mmol/gDW/h")
```

Expected output: a single flux value representing aggregate throughput of the Reactome cholesterol biosynthesis pathway. The leaf-level layer ensures each reaction is counted once.

Example 2 — Pathway-level perturbation analysis (gene knockout → pathway flux change)

Scenario: simulate knockout of HMGCR (HMG-CoA reductase, the statin target) and report which Reactome pathways show the largest flux reduction.

> **When to use this: drug target metabolic impact analysis; identifying pathway-level collateral effects of gene deletion; comparing perturbation signatures across conditions.**
>
> Use leaf-level annotation for flux comparison to avoid double-counting. Use hierarchical annotation if you want to aggregate the result up to top-level pathway categories for reporting.

```
# Knockout HMGCR and compute pathway flux changes
with model:
    model.reactions.get_by_id("EX_glc__D_e").lower_bound = -10
    wt_sol = model.optimize()
    wt_flux = {r: wt_sol.fluxes.get(r, 0) for r in leaf_map}

    # Apply HMGCR knockout via GPR
    ko_result = cobra.flux_analysis.single_gene_deletion(
        model, gene_list=["HMGCR"])

# Aggregate knockout-induced flux changes by Reactome pathway
pathway_delta = {}
for rxn_id, pathway_ids in leaf_map.items():
    delta = abs(ko_result.fluxes.get(rxn_id, 0)) - abs(wt_flux.get(rxn_id,
0))
    for pid in pathway_ids:
        pathway_delta[pid] = pathway_delta.get(pid, 0) + delta

# Report top 10 most affected pathways
import pandas as pd
df = pd.DataFrame(list(pathway_delta.items()),
                  columns=["ReactomeID", "flux_delta"])
print(df.nsmallest(10, "flux_delta"))  # most reduced pathways
```

Expected output: ranked list of Reactome pathway IDs with flux reduction after HMGCR knockout. Pathway IDs can be resolved to names via the Reactome stable identifier API or the ReactomePathways.txt file supplied with Supplementary Data S4.

Example 3 — Pathway enrichment analysis input (hierarchical layer)

Scenario: after transcriptomic integration (e.g. tINIT context-specific model extraction), identify which Reactome pathways are enriched in the set of reactions with significantly altered flux.

When to use this: multi-omics integration; identifying pathway-level signatures from transcriptomics or proteomics data; input to ReactomePA or g:Profiler for gene-set enrichment.

Use the gene-to-pathway mapping (Supplementary Data S4) rather than the reaction-to-pathway mapping, because standard GSEA tools operate on gene lists, not reaction lists. The reaction → GPR → gene → Reactome chain is provided in Supplementary Data S4.

```
# Extract gene set from altered reactions and run Reactome enrichment
# (R code — uses ReactomePA Bioconductor package)

library(ReactomePA)
library(clusterProfiler)

# Load EHMN 2026 gene-to-Reactome mapping (Supplementary Data S4)
gene_reactome_map <- read.csv("ehmn2026_gene_reactome_map.csv")

# Assume: differentially active genes from tINIT extraction
active_genes <- c("HMGCR", "SQLE", "LSS", "DHCR7", "CYP51A1")  # example

# Convert HGNC symbols to Entrez IDs for ReactomePA
entrez_ids <- bitr(active_genes, fromType="SYMBOL",
                   toType="ENTREZID", OrgDb="org.Hs.eg.db")$ENTREZID

# Run Reactome pathway enrichment
enrich_result <- enrichPathway(gene=entrez_ids, organism="human",
                               pvalueCutoff=0.05)
dotplot(enrich_result, showCategory=15)
```

Expected output: dotplot of enriched Reactome pathways. The HGNC symbols in EHMN 2026's FBC geneProduct encoding map directly to the SYMBOL type in bitr without translation.

Table S7.1 — Annotation Tiers and When to Use Each

Annotation tier	What it contains	Typical IDs per reaction	When to use this layer
**All-levels hierarchical (used in Fig. 2B, main statistics)**	All Reactome pathway IDs assigned to a reaction at every level of the hierarchy, from top-level ("Metabolism of lipids") down to the specific event. Includes parent pathway IDs.	3–15 IDs per annotated reaction (parent + child pathways)	Broad biological categorisation; enrichment analysis across subsystems; reporting what fraction of reactions belong to "Metabolism" or "Signal Transduction" parent classes; heat-map visualisation of

			pathway-level flux perturbations at subsystem granularity.
**Leaf-level only (used for quantitative pathway statistics)**	Terminal Reactome event IDs only — pathways with no child nodes in ReactomePathwaysRelation.txt. Eliminates hierarchical duplication.	1–3 IDs per annotated reaction	Quantitative pathway-level flux summation where each reaction should be counted once; perturbation analysis at specific biochemical event level; drug target → pathway impact where event-level precision is needed; input to Reactome pathway analysis tools (ReactomePA R package, g:Profiler) that expect non-redundant gene/reaction lists.
**Reaction-to-gene-to-pathway (derived mapping, Supplementary Data S4)**	ENSG gene → Reactome pathway mapping derived from GPR rules. Allows reaction-level annotation to be projected back to gene space for gene-set enrichment analysis (GSEA).	1–N pathways per gene (all hierarchy levels)	Gene-set enrichment analysis (GSEA, GSVA); pathway-level differential expression integration; transcription factor → pathway → metabolic flux coupling via TRANSFAC/TRANSPATH; drug target → gene → pathway → flux impact chain.

Table S7.2 — Reactome Coverage Limitations by Reaction Class

Reaction class	Coverage in EHMN 2026	Reactome limitation	Practical implication for users
**Transport reactions (SLC, ABC, facilitated diffusion)**	1,427 reactions; Reactome IDs absent for most individual transport variants	Reactome annotates transport events at the process level (e.g. "SLC-mediated transmembrane transport") rather than individual transporter isoforms. Individual SLC variant reactions are not assigned specific Reactome event IDs.	Transport reactions should be excluded from reaction-level Reactome enrichment analysis. For transporter gene-level analysis, use the gene → Reactome pathway mapping (Supplementary Data S4) directly rather than the reaction-level annotation layer.
**Fatty acid / sphingolipid chain-length variants**	855 chain-length-specific reactions; Reactome annotates the canonical reaction class, not every chain-length variant	Reactome represents "fatty acid beta-oxidation" as a pathway event, not as separate reactions for C6, C8, C10… chain lengths. Chain-length-specific EHMN 2026	For lipid pathway-level analysis, use all-levels hierarchical annotation and aggregate at the parent pathway level (e.g. "R-HSA-77289: Mitochondrial Fatty Acid Beta-Oxidation"). Leaf-level summation will undercount lipid pathway flux if chain-length variants are included.

		reactions share a Reactome parent ID but not individual leaf IDs.	
**Generic / lumped reactions (R*, RE* prefix, legacy KEGG)**	3,514 reactions; limited Reactome coverage because reactions are not yet mapped to specific human biochemical events	Reactome requires a specific, characterised human biochemical reaction to assign an event ID. Generic KEGG-lineage reactions that represent reaction classes rather than specific events cannot be matched.	Legacy R*/RE* reactions are excluded from Reactome-based pathway analysis. Their flux can be interpreted at the subsystem level using the reaction prefix classification (Section 3.1 naming convention) but not at Reactome event level.
**Exchange / sink / demand reactions**	6,476 boundary reactions; zero Reactome IDs by design	Reactome does not represent computational boundary constraints. These are modelling artefacts, not biological events.	Always exclude exchange, sink, and demand reactions from pathway annotation queries. Filter on reaction prefix: EX_* (exchange), SINK_*, DM_* (demand) should be removed before any Reactome analysis.
**Confirmed human reactions outside Reactome curation scope**	159 reactions (e.g. xylose oxidoreductase, minor NMP kinases)	Reactome curation is incomplete for certain metabolic branches, particularly uncommon metabolites and minor isoforms. These reactions have GPR and metabolite annotations but no current Reactome event ID.	These 159 reactions are flagged as Reactome submission candidates. For current analyses, treat as confirmed human metabolic activity without pathway classification. They do not distort pathway statistics when the MAR enzymatic denominator is used.

† 159 confirmed human reactions outside current Reactome curation scope (e.g. xylose oxidoreductase, minor nucleoside monophosphate kinases) are flagged as Reactome submission candidates. They do not distort pathway statistics when the MAR enzymatic denominator is used.
