# Supplementary material for: EHMN 2026: A Thermodynamically Refined, SBML-Standardised Human Metabolic Network for Genome-Scale Analysis and QSP Integration
Source: Metabolites. 2026 Mar 31;16(4):236. doi: 10.3390/metabo16040236 (PMC13118034; doi:10.3390/metabo16040236)
Supplement: Supplementary file 1 [file metabolites-16-00236-s001.zip › Supplementary S8.pdf]

Supplementary S8

The eight-stage pipeline

1. **Stage 0 — Starting reconstruction: Original EHMN (Ma et al. 2007) imported as structural foundation. All reactions, metabolites, and gene associations ingested into the processing pipeline. No merging with Recon3D or Human1.**
2. **Stage 1 — Identifier harmonisation: Metabolite identifiers reconciled against MetaNetX MNXref v4.3. Four-step disambiguation procedure resolves 612 ambiguous cases. Output: uniform namespace across 14,321 species (83.1% MetaNetX, 73.0% ChEBI coverage). Input databases: KEGG, BiGG, MetaNetX, ChEBI.**
3. **Stage 2 — Reaction deduplication: Canonical stoichiometry normalisation followed by forward-reverse duplicate detection. 644 reactions removed or disabled; 22,640 retained. No GPR rules lost. Decision point: compartment-specific reactions preserved even if stoichiometrically identical across compartments.**
4. **Stage 3 — GPR reconstruction and harmonisation: 7,812 GPR rules transferred from Human-GEM; 1,826 additional associations reconstructed. Gene IDs standardised to HGNC symbols and ENSG IDs. All encoded in SBML FBC2 geneProductAssociation Boolean format. Input databases: Human-GEM, HGNC, Ensembl.**
5. **Stage 4 — Thermodynamic directionality refinement: Phase 1: 1,923 reactions re-constrained by biochemical irreversibility rules (validated against KEGG, BRENDA, MetaCyc). Phase 2: boundary-closure LP detects and eliminates 37 infeasible cycles iteratively. Phase 3: flux consistency analysis confirms 0 remaining loops. Decision point: near-equilibrium reactions retained as reversible unless structural evidence supports constraint.**
6. **Stage 5 — Reactome pathway mapping: ENSG IDs from GPR rules mapped to Reactome release 88. Two annotation layers constructed: all-levels hierarchical and leaf-only. 2,193 unique pathway IDs assigned across 7,910 reactions. Input: Ensembl2Reactome_All_Levels.txt, ReactomePathwaysRelation.txt.**
7. **Stage 6 — Chemical consistency validation: Formula and charge verified for all species. Hub metabolite cross-check (13 cofactor pools in all primary compartments). Species without formulas categorised by class. Decision point: ambiguous ChEBI mappings not written to SBML to avoid incorrect cross-references.**
8. **Stage 7 — SBML encoding and validation: Full SBML Level 3 Version 2 + FBC2 encoding. libSBML consistency checks: structural validity, FBC syntax, unique identifiers, no orphan references. Output: EHMN_2026_repaired_pipeline_SBML_L3V2.xml.2**

Supplementary Figure S8.1. Detailed eight-stage reconstruction and refinement workflow for EHMN 2026. Each stage is shown with its input databases/resources (left), key computational actions and decision points (centre), and quantitative outputs (right). Downward arrows indicate sequential data flow; decision diamonds indicate branching steps with explicit resolution rules. Stage colour coding corresponds to the primary analytical function: blue (identifier harmonisation), dark green (structural consolidation), dark gold (gene annotation), dark red (thermodynamic refinement), purple (pathway mapping), teal (chemical validation), navy (standards encoding). All input database versions and roles are listed in Supplementary Table S8.1

Supplementary Table S8.1— Workflow Stage Details

Stage	Stage name	Input(s)	Output / key metric	What happens — decision points and tools
0	**Starting reconstruction**	Original EHMN (Ma et al. 2007, Mol Syst Biol 3:135)	**Baseline: reactions, metabolites, legacy IDs**	Import all reactions, metabolites, and gene associations from the 2007 EHMN into a structured processing pipeline. No merging with Recon3D or Human1; EHMN lineage preserved throughout.
1	**Identifier harmonisation (Section 2.2)**	KEGG, BiGG, legacy EHMN IDs; MetaNetX v4.3 cross-reference tables; ChEBI ontology	**83.1% MetaNetX coverage; 73.0% ChEBI-validated; 612 ambiguous mappings resolved; 430 redundant entries removed**	4-step procedure: (i) exact ID matching; (ii) InChIKey/formula-supported matching; (iii) stoichiometric consistency check across candidate mappings; (iv) rule-based disambiguation for 612 ambiguous cases (protonation state, stereochemistry, compartment). Output: uniform MNXref namespace across all 14,321 species.
2	**Reaction deduplication (Section 2.2.2)**	Harmonised reaction set (23,284 pre-dedup reactions)	**644 reactions removed or disabled; 22,640 reactions retained; 0 GPR rules lost**	3-step procedure: (i) canonical stoichiometry normalisation (alphabetically sorted, normalised coefficients, proton-only differences removed); (ii) forward-reverse duplicate detection; (iii) compartment-specific validation. 438 reverse-direction duplicates and 206 exact duplicates

				identified. 214 retained but disabled for traceability.
3	**GPR reconstruction & harmonisation (Section 2.3)**	Human-GEM reaction-gene matrix; HGNC, Ensembl, UniProt cross-references	**9,638 reactions with GPR; 3,996 gene products; 2,887 unique ENSG IDs**	Gene identifiers standardised to HGNC symbols and ENSG IDs. 7,812 GPR rules transferred from Human-GEM; 1,826 additional associations reconstructed via reaction-gene mapping. Ambiguous reactions assigned OR-only associations. Boolean GPR encoded in SBML FBC2 geneProductAssociation format.
4	**Thermodynamic directionality refinement (Section 2.5)**	Deduplicated reaction set; KEGG/BRENDA/MetaCyc reaction annotations; biochemical irreversibility rules	**1,923 reactions re-constrained; 37 infeasible cycles resolved; 0 remaining loops; 9,792 irreversible reactions (43.2%)**	Phase 1: apply biochemical irreversibility rules (ATP ligases, decarboxylations, NADH/NADPH reductions, OXPHOS/ETC, beta-oxidation) to 1,923 reactions. Phase 2: boundary-closure LP detects all reactions sustaining non-zero flux without external substrates; iterative passes eliminate 37 infeasible cycles. Phase 3: flux consistency analysis confirms 0 remaining unconstrained ATP-generating cycles.
5	**Reactome pathway mapping (Section 2.4)**	Ensembl2Reactome_All_Levels.txt (Reactome release 88); GPR-derived ENSG IDs	**7,910 reactions annotated (34.9% overall; 61% of MAR enzymatic core); 2,193 unique pathway IDs; 642 leaf-level events**	ENSG IDs from GPR rules mapped to Reactome pathways. Two layers built: (i) all-levels hierarchical (all parent + child IDs per reaction); (ii) leaf-only (terminal events, ReactomePathwaysRelation.txt used to identify nodes with no children). 76% of 2,887 ENSG

				IDs successfully mapped; 24% unmapped due to incomplete Reactome annotation or obsolete identifiers.
**6**	**Chemical consistency validation (Section 2.7 / Supp. Q2)**	Species formula/charge from pipeline; hub metabolite list	**61.2% formula coverage; 73.0% ChEBI coverage; all 13 hub cofactors fully annotated in primary compartments; 0 critical formula errors in MAR core**	Formula and charge annotation verified for all species. Hub metabolite cross-check: ATP, ADP, AMP, NAD+, NADH, NADP+, NADPH, CoA, CO2, H2O, Pi, PPi, H+ confirmed in all primary compartments. Species without formula categorised by class (protein pools, uncertain compartment, complex lipids, generic redox handles, specific metabolites awaiting curation).
**7**	**SBML encoding and validation (Section 2.6)**	Final harmonised reconstruction	**EHMN_2026_repaired_pipeline_SBML_L3V2.xml; libSBML validation: PASSED; 0 orphan references**	Encoded in SBML Level 3 Version 2 with FBC2 package. Explicit compartment definitions, species, reactions, gene products, reaction bounds. libSBML consistency checks: structural validity, correct FBC syntax, unique identifiers, no orphan references, no embedded kinetic laws. Output: standards-compliant SBML for COBRApy, COBRA Toolbox, Tellurium.

Table S8.1 Detailed workflow stage specifications for the EHMN 2026 reconstruction pipeline. For each of the eight pipeline stages, the table provides: input databases/resources, key computational actions and decision points, key quantitative output metrics, and the section of the main manuscript where the stage is described. This table provides the text-reproducible equivalent of Supplementary Figure S1

Supplementary Table S8.2 — Input Database Registry

Database / resource	Version / release used	Stages that use it	Role in pipeline
**Original EHMN (Ma et al. 2007)**	Published 2007; Mol Syst Biol 3:135	Stage 0	Structural foundation; all reactions, metabolites, and gene associations imported as starting point
**MetaNetX / MNXref**	v4.3 (release 2023-05)	Stage 1 (primary)	Metabolite identifier reconciliation; cross-reference tables for KEGG, ChEBI, Rhea, BiGG, MetaCyc
**ChEBI**	Current as of 2023-05	Stage 1, Stage 6	Chemical formula, charge, and ontology validation; 10,448 metabolites ChEBI-validated
**KEGG Reaction database**	Current as of 2023	Stage 1, Stage 4	Legacy identifier source; reaction directionality annotations for thermodynamic curation
**Human-GEM**	Current at time of construction	Stage 3 (GPR source)	7,812 GPR rules transferred; reaction-gene matrix used for GPR reconstruction
**HGNC / Ensembl**	Current as of 2023	Stage 3	Gene identifier standardisation; HGNC symbols and ENSG IDs for all 3,996 gene products
**BRENDA enzyme database**	Current as of 2023	Stage 4	Validation of irreversibility assignments for ATP ligases, redox reactions, beta-oxidation
**MetaCyc**	Current as of 2023	Stage 4	Validation of decarboxylation and energy-currency reaction directionality
**Reactome**	Release 88 (2023)	Stage 5	Pathway annotation; Ensembl2Reactome_All_Levels.txt and ReactomePathwaysRelation.txt
**libSBML**	Current stable at encoding	Stage 7	SBML Level 3 + FBC2 encoding and validation; consistency checks

Table S8.2 Registry of all external databases and computational resources used in the EHMN 2026 reconstruction pipeline, with version/release dates, pipeline stages that use each resource, and functional role. Provides the complete information required to reproduce the identifier harmonisation, GPR reconstruction, thermodynamic curation, and pathway annotation steps."
