# Supplementary material for: EHMN 2026: A Thermodynamically Refined, SBML-Standardised Human Metabolic Network for Genome-Scale Analysis and QSP Integration
Source: Metabolites. 2026 Mar 31;16(4):236. doi: 10.3390/metabo16040236 (PMC13118034; doi:10.3390/metabo16040236)
Supplement: Supplementary file 1 [file metabolites-16-00236-s001.zip › Supplementary S9.pdf]

Supplementary S9

2.1 Size as a reflection of modelling approach, not accuracy

The reviewer is correct that the current manuscript — particularly the "+68% reactions, +91% metabolites" framing in Table 1 and Section 4.1 — could be read as implying that larger is better. This framing is misleading in two directions: it overstates EHMN 2026's claim (the extra reactions are boundary conditions and lipid variants, not additional enzymatic knowledge) and it understates its genuine contributions (which are in annotation quality, not reaction count).

Table S9.1 provides a feature-by-feature decomposition of each metric difference, classifying each as "reconstruction scope" (size reflects approach), "intentional design choice" (extra detail that serves a specific purpose), or "genuine quality difference" (annotation improvement independent of size). This analysis shows:

- **Reaction count (+68%):** reflects reconstruction scope — extra boundary and transport reactions, not extra enzymatic knowledge. Not a quality claim.
- **Metabolite count (+91%):** reflects intentional design choice — 11 compartments and chain-length lipid variants for granular lipid analysis. Extra detail with a specific purpose.
- **GPR coverage (42.6% vs ~97%):** reflects reconstruction architecture — larger boundary set in the denominator. Not a quality deficit; 62% on the enzymatic core. GPR completeness was not a primary goal.
- **Thermodynamic irreversibility (43.2% vs ~35–40%):** a genuine quality difference — more reactions correctly constrained, 37 futile cycles eliminated. This is the primary analytical quality claim.
- **Reactome coverage (61% enzymatic core vs ~15–31%):** a genuine quality difference — systematic annotation enabling pathway-level analysis not available in either comparator.

2.2 Specific dimensions of intentional extra detail

Table S9.2 consolidates the three dimensions where EHMN 2026 intentionally offers more detail than Human1 and Recon3D, with biological and computational rationale for each. In brief:

1. **Compartmental granularity:** 11 compartments including explicit inner mitochondrial membrane (OXPHOS proton gradient), peroxisome (very-long-chain FA β-oxidation), and lysosome — compartments that enable reactions not representable in models without this spatial resolution.
2. **Lipid chain-length variants:** 855 FA/sphingolipid reactions at individual chain-length resolution (C6–C26). This enables lipidomics data integration and lipotoxicity modelling at a granularity not available in Human1 or Recon3D.
3. **Annotation architecture:** uniform MetaNetX namespace + Reactome event IDs per reaction + HGNC/ENSG gene encoding simultaneously in one SBML file. This combination is not available in either comparator in their current deposited forms.

2.3 GPR completeness is not a primary goal

The current manuscript states this implicitly in Section 4.6 ("the comparatively lower GPR coverage relative to Recon3D and Human1 should not be interpreted as reduced annotation quality") but does not state it plainly and early. The revised Section 4.2 text adds an explicit statement in the Discussion where it will be most visible to readers coming from the comparison tables.

3. Table S9.1 — Size vs Quality Decomposition

Proposed as Table 10 in revised Section 4.2 (see Change 2, Section 5). Colour coding in the final column: grey = scope, blue = design choice, red = architecture, green = genuine quality gain.

Feature / metric	EHMN 2026	Human1 / Recon3D	What the difference reflects — size or quality?
**Total reactions (22,642 vs ~13,400)**	**22,642**	~13,400–13,543	RECONSTRUCTION SCOPE, not accuracy. The additional ~9,200 reactions are boundary conditions (exchange, sink, demand: 6,476), transport isoforms (1,427), and chain-length lipid variants (855) required for stoichiometric solvability and metabolite granularity. They do not represent additional enzymatic knowledge over Human1/Recon3D.
**Metabolites / species (14,321 vs ~4,000–8,000)**	**14,321**	~4,140–8,378	INTENTIONAL DESIGN CHOICE providing extra detail. 11 compartments vs 10–13; explicit representation of inner mitochondrial membrane (20 species), peroxisome (844), and lysosome (640) as distinct compartments; FA chain-length variants as separate species (e.g. C6:0, C8:0, C10:0 distinguished in β-oxidation). This enables more granular lipid pathway flux analysis than either comparator.
**Gene products (3,996 vs 3,288–3,628)**	**3,996**	3,288–3,628	ANNOTATION BREADTH, not inflated. EHMN 2026 exceeds both comparators despite conservative GPR philosophy. The larger gene count reflects the larger MAR enzymatic core (12,969 reactions), not speculative gene attribution.
**GPR coverage overall (42.6% vs ~96–97%)**	**42.6%**	~96–97%	RECONSTRUCTION ARCHITECTURE, not a quality gap. Human1/Recon3D's near-complete GPR is computed over a smaller denominator that excludes most boundary reactions. EHMN 2026 retains a larger boundary reaction set explicitly; restricting to the MAR enzymatic core gives 62% GPR — the

			like-for-like comparison. GPR completeness was explicitly not a primary goal of EHMN 2026 (see Section 4.2).
**Thermodynamic irreversibility (43.2% vs ~35–40%)**	**43.2%**	~35–40%	QUALITY DIFFERENCE — intentional. EHMN 2026 has more irreversible reactions because 1,923 reactions were explicitly re-constrained based on biochemical evidence and 37 futile cycles were eliminated. This is a genuine analytical quality improvement: it eliminates spurious reverse-flux solutions that distort FBA predictions in the comparators.
**Reactome pathway coverage (61% enzymatic core vs ~15–31%)**	**61%**	~15–31%	QUALITY DIFFERENCE — intentional. EHMN 2026's systematic Reactome annotation (2,193 unique IDs across 7,910 reactions) enables pathway-level flux analysis that neither Human1 nor Recon3D supports natively. This is an annotation quality gain, not a size effect.
**MetaNetX identifier namespace (83.1% coverage)**	**83.1%**	Not systematically applied	QUALITY DIFFERENCE — intentional. Uniform MNXref namespace enables namespace-clean integration with external tools and QSP frameworks. This is an annotation quality gain independent of model size.

4. Table S9.2 — Dimensions of Intentional Extra Detail

Proposed as Table 11 in revised Section 4.2, immediately after Table 10.

Dimension of extra detail	What EHMN 2026 provides	Biological / computational benefit over Human1 and Recon3D
**Metabolite granularity — compartments**	11 compartments including explicit inner mitochondrial membrane (20 species), peroxisome (844 species), lysosome (640 species), and uncertain compartment (1,858 species flagged for curation)	Inner mitochondrial membrane compartment enables explicit representation of OXPHOS proton gradient reactions, ATP synthetase directionality, and NADH shuttle stoichiometry — not separately representable in models without this compartment. Peroxisome compartment enables explicit very-long-chain FA β-oxidation separate from mitochondrial β-oxidation.
**Metabolite granularity — lipid chain lengths**	855 fatty acid and sphingolipid chain-length variant reactions representing individual chain lengths (C6, C8, C10, C12, C14, C16, C18, C20, C22, C24, C26) as distinct species and reactions	Enables quantitative lipid flux analysis at chain-length resolution — e.g. distinguishing C16:0 vs C18:0 palmitoyl-CoA flux through β-oxidation, or ceramide vs sphingomyelin chain-length distribution. This granularity is

		required for lipidomics data integration and lipotoxicity modelling; neither Human1 nor Recon3D represent chain-length variants as distinct reactions.
**Annotation architecture — namespace coherence**	Uniform MetaNetX MNXref namespace across 83.1% of 14,321 species; ChEBI-validated for 73.0%; Reactome event IDs per reaction for 7,910 reactions (61% of enzymatic core); all gene products in HGNC/ENSG	Namespace-clean integration with any downstream tool (pathway databases, QSP frameworks, transcriptomics pipelines) without ID translation. The three annotation layers (metabolite: MNXref; reaction: Reactome; gene: HGNC/ENSG) are simultaneously available in the deposited SBML — a property not shared by either comparator in its current publicly deposited form.
