# Supplementary material for: EHMN 2026: A Thermodynamically Refined, SBML-Standardised Human Metabolic Network for Genome-Scale Analysis and QSP Integration
Source: Metabolites. 2026 Mar 31;16(4):236. doi: 10.3390/metabo16040236 (PMC13118034; doi:10.3390/metabo16040236)
Supplement: Supplementary file 1 [file metabolites-16-00236-s001.zip › Supplementary Table S5.pdf]

Supplementary Table S5

"Validation basis for the 1,923 reactions assigned directionality constraints during thermodynamic refinement in EHMN 2026. Reaction classes, approximate counts, biochemical basis for irreversibility, and representative examples with external database evidence. Validation sources: KEGG Reaction database; BRENDA enzyme database; MetaCyc metabolic pathway database; Human-GEM source annotations (Robinson et al. 2020). eQuilibrator ΔG′° estimates at pH 7.4, T = 310 K, ionic strength 0.15 M (Noor et al. 2013)."

Validation of the 1,923 Re-constrained Reactions

Supplementary Table S5 (see Change 2, Section 6).

Reaction class	~Count	Validation basis	Representative reactions & evidence
**ATP-dependent ligases (biosynthetic reactions)**	**~420**	ATP hydrolysis thermodynamics: ΔG′° of ATP hydrolysis −30.5 kJ/mol at pH 7; biosynthetic coupling makes overall reaction strongly negative. Verified: KEGG, BRENDA.	Fatty acid CoA synthetases (ACSL1–6); aminoacyl-tRNA synthetases; acetyl-CoA carboxylase. BRENDA enzyme data confirm irreversibility for all human isoforms.
**Decarboxylation reactions**	**~310**	CO_2 loss renders reaction irreversible under biological concentrations (ΔG′° typically −20 to −40 kJ/mol). Verified: MetaCyc reaction database.	Pyruvate dehydrogenase (MAR04391); isocitrate dehydrogenase CO_2 step; branched-chain α-keto acid decarboxylases (BCKDHA/BCKDHB). MetaCyc confirms irreversibility for all.
**NADH/NADPH-coupled reductions (large negative ΔE°)**	**~380**	Standard reduction potential difference ΔE° > +100 mV; physiological $[NADH]/[NAD^+]$ and $[NADPH]/[NADP^+]$ ratios enforce directionality. eQuilibrator estimates ΔG′° < −30 kJ/mol at pH 7.4.	Glutamate dehydrogenase (GDH1/2); thioredoxin reductase (TXNRD1/2); dihydrofolate reductase (DHFR). eQuilibrator component contribution values confirm assignment.
**OXPHOS / ETC complexes**	**~20**	Proton-motive-force driven: in vivo ΔΨ ~−180 mV and ΔpH	Complex I (NDUF subunits); Complex III (UQCR); Complex IV (COX); ATP synthase (ATP5). Literature: Mitchell

		~0.5–1 units enforce directionality. 80% irreversibility in EHMN 2026 matches standard OXPHOS physiology.	(1961); Nicholls & Ferguson Bioenergetics (2013, 4th ed.).
**Fatty acid β-oxidation spiral**	**~290**	Net ΔG′° ~−69 kJ/mol per cycle under physiological conditions; acyl-CoA thioester activation commits forward directionality. Verified: BRENDA, Human1 annotations.	ACADM, ACADL, ACADS (acyl-CoA dehydrogenases); HADHA/HADHB (trifunctional protein); ACAA2 (thiolase). EHMN 2026 achieves 59.1% irreversibility in this class.
**Other energy-currency reactions (phospho-group transfers, sulfation, etc.)**	**~503**	Directionality consistent with source Human-GEM annotations and MetaCyc reaction database; cross-checked where ΔG′° literature values confirm net negative ΔG.	Nucleoside diphosphate kinases; sulfotransferases (SULT1A1 etc.); PRPP synthetase. Human-GEM GPR rules and MetaCyc used as validation cross-reference.

† Counts are approximate class estimates. Exact per-reaction assignment is available in Supplementary Data S3 (bounds table). All five classes are exhaustive — every re-constrained reaction falls into one category.

5. Summary of Manuscript Changes

#	Change	Location	What it adds
1	**Expand Section 2.5 (~200 → ~350 words)**	Section 2.5, replace entire section	Adds: explicit reasoning for approach choice; comparison with llFBA/TFBA/eQuilibrator; statement that 1,923 reactions validated against KEGG/BRENDA/MetaCyc; roadmap for ΔG′° extension
2	**Add Supplementary Table S5**	New Supplementary Table S5	Provides reaction-class breakdown with validation basis for all 1,923 re-constrained reactions — directly addresses reviewer request
3	**Add one sentence to Section 2.5 after metric table**	Section 2.5, after existing metric table	Points readers to Supplementary Table S5 for validation details

4	**Expand Section 4.3 (Discussion) with future roadmap**	Section 4.3, new paragraph at end	States two-step plan for full $\Delta G'^{\circ}$ extension (formula completion then concentration data from CYTOCON DB); closes the "postponed" loop the reviewer flagged
5	**Insert Table 5 (approach comparison) in Section 3.4**	Section 3.4, after key-finding paragraph	Puts semi-quantitative vs full ΔG comparison in Results where it is most visible to readers
