# Supplementary material for: EHMN 2026: A Thermodynamically Refined, SBML-Standardised Human Metabolic Network for Genome-Scale Analysis and QSP Integration
Source: Metabolites. 2026 Mar 31;16(4):236. doi: 10.3390/metabo16040236 (PMC13118034; doi:10.3390/metabo16040236)
Supplement: Supplementary file 1 [file metabolites-16-00236-s001.zip › Supplementary_S1.pdf]

Supplementary Data S1

Model Structure and Core SBML Contents (EHMN_2026)

This supplementary document describes the structural components of the final EHMN_2026 genome-scale reconstruction. The SBML model is encoded in Level 3 Version 2 with the FBC extension and represents the fully harmonised, deduplicated, and thermodynamically refined version of the network.

S1.1 Final Model Statistics

Metric	Value
Compartments	11
Metabolites (species)	14,321
Reactions	22,640
Gene products	3,996
Reactions with GPR	9,638 (42.6%)
Unique ENSG identifiers	2,887
Reactome-associated reactions (all levels)	2,194
Reactome-associated reactions (leaf level)	1,278

S1.2 SBML File Contents

The SBML file contains: • Complete reaction list with stoichiometry and reversibility flags. • Gene–protein–reaction (GPR) associations. • Compartment definitions and species assignments. • MetaNetX and ChEBI metabolite cross-references. • Reactome pathway annotations. • Flux bounds consistent with thermodynamic refinement.

S1.3 Reproducibility and Versioning

The model version corresponds to EHMN_2026_ENSGnormalized_reactomeAnnotated_v2. A BioModels accession number will be assigned upon deposition. A mirrored version will be available at www.iqanova.org/atlas with version control and checksum documentation.
